# Systematic Review of Ozone Effects on Human Lung Function, 2013 Through 2020

**DOI:** 10.1016/j.chest.2021.07.2170

**Published:** 2021-08-10

**Authors:** Stephanie M. Holm, John R. Balmes

**Affiliations:** a Division of Epidemiology, School of Public Health, University of California, Berkeley, Berkeley, CA; b Division of Environmental Health Sciences, School of Public Health, University of California, Berkeley, Berkeley, CA; c Western States Pediatric Environmental Health Specialty Unit, University of California, San Francisco, CA; d Division of Occupational and Environmental Medicine, University of California, San Francisco, San Francisco, CA

**Keywords:** adult, air pollution, child, epidemiology, spirometry, EPA, Environmental Protection Agency, ISA, Integrated Science Assessment, NAAQS, National Ambient Air Quality Standards, PEF, peak expiratory flow, ppb, parts per billion

## Abstract

**Background:**

Ozone effects on lung function are particularly important to understand in the context of the air pollution-health outcomes epidemiologic literature, given the complex relationships between ozone and other air pollutants with known lung function effects.

**Research Question:**

What has been learned about the association between ozone exposures and lung function from epidemiology studies published from 2013 through 2020?

**Study Design and Methods:**

On March 18, 2018, and September 8, 2020, PubMed was searched using the terms *health AND ozone*, filtering to articles in English and about humans, from 2013 or later. An additional focused review searching for *ozone AND (lung function OR FEV*_*1*_*OR FVC)* was performed June 26, 2021. Articles were selected for this review if they reported a specific relationship between a lung function outcome and ozone exposure.

**Results:**

Of 3,271 articles screened, 53 ultimately met criteria for inclusion. A systematic review with assessment of potential for bias was conducted, but a meta-analysis was not carried out because of differences in exposure duration and outcome quantification. Consistent evidence exists of small decreases in children’s lung function, even associated with very low levels of short-term ozone exposure. The effects on adult lung function from exposure to low-level, short-term ozone are less clear, although ozone-associated decrements may occur in the elderly. Finally, long-term ozone exposure decreases both lung function and lung function growth in children, although few new studies have examined long-term ozone and lung function in adults.

**Interpretation:**

Much of this literature involves concentrations below the current US Environmental Protection Agency’s National Ambient Air Quality Standard of 70 parts per billion over an 8-h averaging time, suggesting that this current standard may not protect children adequately from ozone-related decrements in lung function.

Ozone is an air pollutant with detrimental effects on lung function. It is generated as a secondary pollutant, involving reactions with nitrogen oxides,^1^ and thus varies with levels of these pollutants. This covariance means that real-world effects of ozone on lung function may be particularly difficult to assess in a single epidemiologic study.

Based on the 2013 US Environmental Protection Agency (EPA) Integrated Science Assessment (ISA)^2^ review of the health effects of ozone, the National Ambient Air Quality Standard (NAAQS) was decreased from 75 to 70 parts per billion (ppb) over an 8-h averaging time. Yet, evidence of health effects at low levels continues to accumulate, raising the possibility of a need for further regulatory action.

Multihour ozone levels of 80 ppb or higher consistently produce significant decreases in lung function in healthy adults.^2^ At the time of the 2013 EPA review, only a handful of studies had assessed lung function in association with ozone exposures of < 80 ppb or for exposure durations longer than several days.^2^ In the 8 years since that review, the Results of multiple epidemiologic studies of the short- and long-term lung function effects of ozone have been reported, many of which involved exposures of less than the current NAAQS.

The EPA released its most recent ISA for ozone in early 2020. Because of regulation limiting the science that could be considered by the federal EPA, the lung function section of that ISA focuses almost exclusively on experimental studies. This article fills the remaining knowledge gap by systematically reviewing the epidemiologic studies.

## Methods

**Figure 1:**
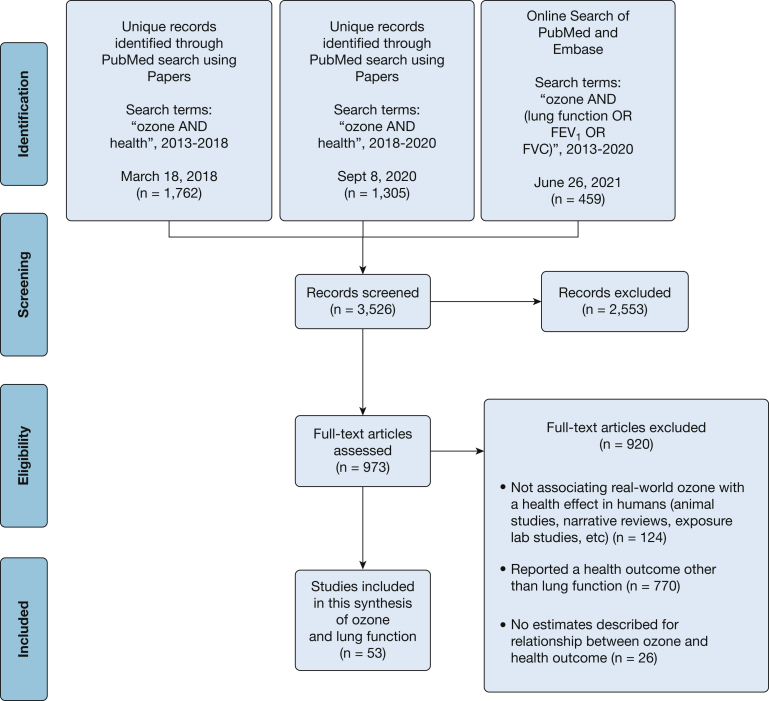
Flow chart showing ozone health effects articles search strategy.

This systematic review follows an initial review^3^ performed to cover all published work on the health effects of ozone in children and adults in the 5 years after the 2013 ISA. On March 18, 2018, we searched PubMed using the terms *health AND ozone*, filtering to articles in English and about humans from 2013 or later. Screening of abstracts and articles was performed by S. M. H. On September 8, 2020, this was repeated. The 2020 search was identical with one exception: articles describing effects on COVID-19 were excluded for consistency. A third search was performed June 26, 2021, in both PubMed and Embase, using the more focused search terms *ozone AND (lung function OR FEV1 OR FVC)*. To be included, an article must have estimated a specific ozone-lung function relationship (such as ozone associated with a difference in FEV_1_), not simply an estimate for a mixture of pollutants that includes ozone, nor for a relationship between ozone and “reduced lung function.” After screening and full-text review, a total of 53 articles were included in this review (Fig 1).

All studies involving real-world ozone exposures were included; only experimental studies and reviews were excluded. Both short- and long-term studies of lung function were reviewed, using the same 1-month cutpoint as defined in the 2013 ISA. Unfortunately, different studies use different ozone metrics (eg, peak 8-h average, peak 12-h average, 24-h average), and no clear way exists to convert between these metrics. Data extraction was performed by S. M. H., and data were entered into a table summarizing the study design, the studied population, method of ozone assessment, outcomes, and other notes (e-Appendix 1).

Primary assessments of study quality were performed by both authors using the Newcastle-Ottawa scale. Each author performed the assessments independently, and disagreements were resolved by consensus. The Newcastle-Ottawa scale was chosen because it is the most frequently used in air pollution systematic reviews and is considered an acceptable bias assessment technique (See e-Appendix 2 for specific adaptations of the scale used in this study).^4^^,^^5^ A secondary bias assessment was performed by S. M. H., using the Risk of Bias In Non-Randomized Studies of Interventions tool on the recommendation of peer reviewers.^6^ Risk-of-bias assessments for all articles are included in e-Appendixes 3 and 4.

Summary measures were described as reported (ORs, risk differences, etc.). Results were scaled linearly per 10 ppb of ozone when appropriate. Results in micrograms per cubed meter were converted with a conversion factor of 2. Where the range of participant ages was not listed, it was assumed to be the reported mean ± 2 times the SD.

We decided against performing meta-analyses because it would be inappropriate to pool estimates from different types of exposure models (multiplicative vs additive), those involving different units of lung function outcomes (absolute volume vs % predicted using standardized populations), and those with different durations of ozone exposure. Instead, Results are plotted separately (for the first two items) and ordered by exposure duration (the third item) so that patterns resulting from exposure duration can be seen. The Preferred Reporting Items in Systematic Reviews and Meta-Analyses checklist was completed (e-Appendix 5).

## Results

### Short-Term Ozone and Lung Function in Children and Adolescents

**Figure 2:**
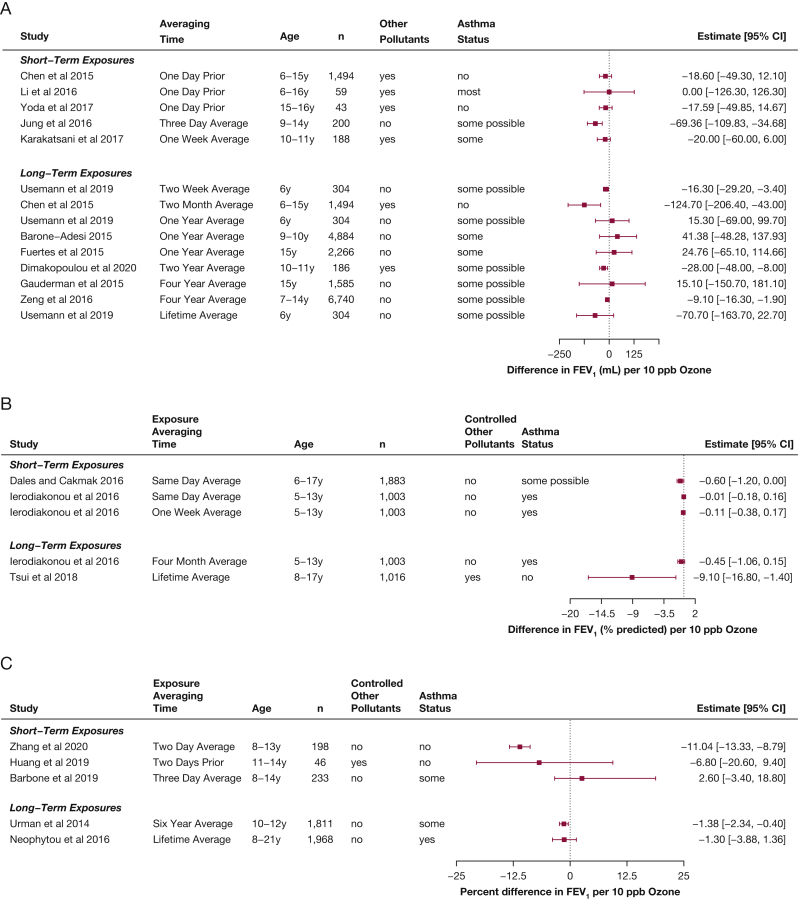
A-C, Forest plots showing estimates of differences in FEV_1_ associated with increases of 10 ppb ozone in children, from studies published from 2013 through 2020 within the studies that reported differences in milliliters (A), within the studies that reported differences in percent predicted (B), and in studies that reported multiplicative changes (C), in percent differences.

In children, new research continues to suggest small decrements in lung function associated with short-term exposures, including to relatively low levels of ozone (mean, 5-56 ppb). Nine of the 11 new studies that assessed the relationship between short-term ozone exposures and FEV_1_ in children demonstrated small decreases associated with increases of 10 ppb of ozone over times ranging from 1 day to 2 weeks (change of 0 to –69 mL, –0.01 to –9% predicted, or +2.6 to 11%), and all showed levels of less than the current NAAQS (Fig 2).^7^, ^8^, ^9^, ^10^, ^11^, ^12^, ^13^, ^14^, ^15^, ^16^, ^17^ Although the CIs for some estimated effects crossed the null, the relative consistency in findings suggested that increases in ozone concentration, even at low levels, are associated with decreases in FEV_1_ in healthy children, although it may be a relatively small effect. Limitations of these studies include lack of copollutant control in nearly half of the studies and lack of control for socioeconomic status, as well as exposure misclassification resulting from the use of measurements from fixed outdoor locations; the studies by Karakatsani et al^8^ and Dimakopoulou et al^18^ are the only studies reported during the review period that used personal ozone monitoring. The three studies rated as having the lowest risk of bias among this group^19^, ^20^, ^21^ all had point estimates with larger associated decreases (–2% to –42%).

**Figure 3:**
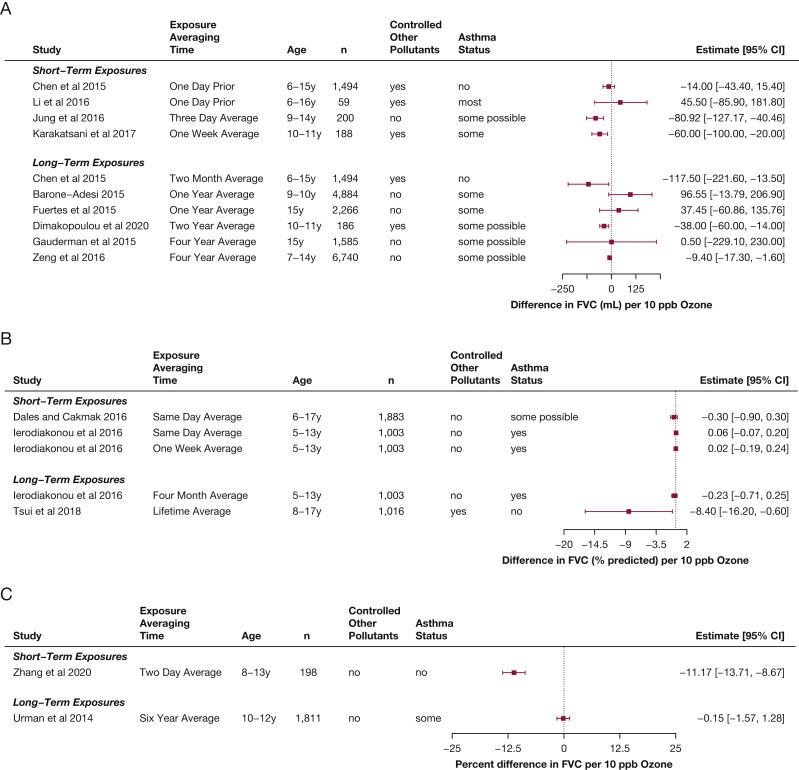
A-C, Forest plots showing estimates of differences in FVC associated with increases of 10 ppb ozone in children, from studies published from 2013 through 2020 within the studies that reported differences in milliliters (A), within the studies that reported differences in percent predicted (B), and in studies that reported multiplicative changes (C), in percent differences.

Measurement of FVC can be more difficult to obtain than FEV_1_ in children (because of the need for a more sustained exhalation), and fewer studies reported short-term FVC effects for children (Fig 3).^7^^,^^8^^,^^11^^,^^14^^,^^15^^,^^19^^,^^21^ However, generally consistent small decreases in FVC in children and adolescents were found, associated with short-term ozone exposures (–14 to –81 mL per 10 ppb ozone, –0.3% predicted, or a –11% difference).

**Figure 4:**
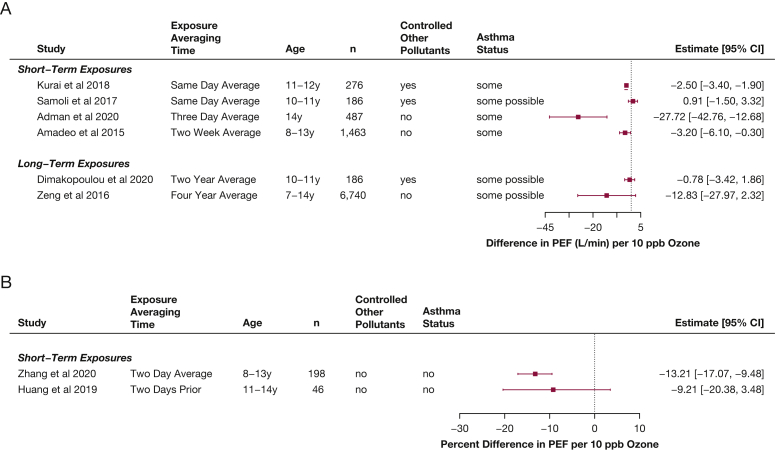
A-B, Forest plots showing estimates of differences in PEF associated with increases of 10 ppb ozone in children, from studies published from 2013 through 2020 within the studies that reported differences in milliliters (A) and in studies that reported multiplicative changes (B), in percent differences. PEF = peak expiratory flow.

Peak expiratory flow (PEF) is used sometimes to recognize exacerbations in children with asthma.^22^ Some additional studies have used PEF to assess the association between short-term ozone exposures and airways obstruction in healthy children (Fig 4)^19^^,^^20^^,^^23^, ^24^, ^25^, ^26^: all but one estimated that PEF decreased as ambient ozone concentration increased (–28 to 1 L/min or –13% to –9% difference). An additional study that measured oxidants (but thought mostly to represent ozone) also found a 0.26% decrease in PEF for healthy children associated with increases in oxidants (95% CI, –0.49 to –0.03).^27^ Although PEF often is criticized as more sensitive to participant effort, these findings are very consistent with those demonstrating decreases in FEV_1_.

Some subpopulations of children may be at increased risk of ozone-related lung function decrements after short-term exposures. Certain genotypes of the *SOD2 V16A* gene seem to interact with ozone exposures to produce significant differences in forced expiratory flow at 25th to 75th percent of the FVC.^28^ Another study found a small, nonsignificant decrease in forced expiratory flow at 25th to 75th percent of the FVC in children with asthma,^29^ again with a gene by environment effect; participants with no *GSTM1* gene product showed a significant decrease of 6 mL/s per 10 ppb of ozone. Findings are conflicting regarding whether the relationship between lung function and ozone exposure may be stronger in girls^30^ or boys.^31^ Another study suggested that findings were larger among those who were not breastfed as infants,^32^ a third study found that effects are larger in youths with mood disorders^15^ and a fourth study found larger effects of ozone exposure in children who were overweight or obese.^33^

The five studies that assessed the ozone-lung function relationship among children with asthma seemed to demonstrate smaller, null, or occasionally inverse relationships between ozone and FEV_1_ and FVC.^11^^,^^17^^,^^21^^,^^34^ Ierodiakanou et al^11^ and Li et al^17^ both adjusted for medication use, Benka-Coker et al^34^ adjusted for asthma severity (a proxy for medication use), and all participants in the study by Hernandez et al^21^ were receiving long-term inhaled corticosteroids. Given the small or null findings in the studies of children with asthma, the use of inhaled corticosteroids by many of these children may have decreased responsiveness to ozone, resulting in some residual confounding. The findings from the Benka-Coker et al^34^ study were not included in the figures because that study used *Z* scores. Two additional studies were published in the review interval that described ozone-lung function Results in children, but were not included in the figures or description above because the results for ozone exposure had been published previously^35^ or because the results described in the article were not internally consistent.^36^

Overall, small but consistent decreases in lung function associated with short-term ozone exposure seem to occur in healthy children, including at low levels. These effects are less strong in children with asthma, and we hypothesize that this may be the result of amelioration of the inflammatory response by inhaled corticosteroids. However, recent evidence suggests that particular genotypes may increase susceptibility to ozone, making certain children at particularly high risk.

### Short-Term Ozone and Lung Function in Adults

**Figure 5:**
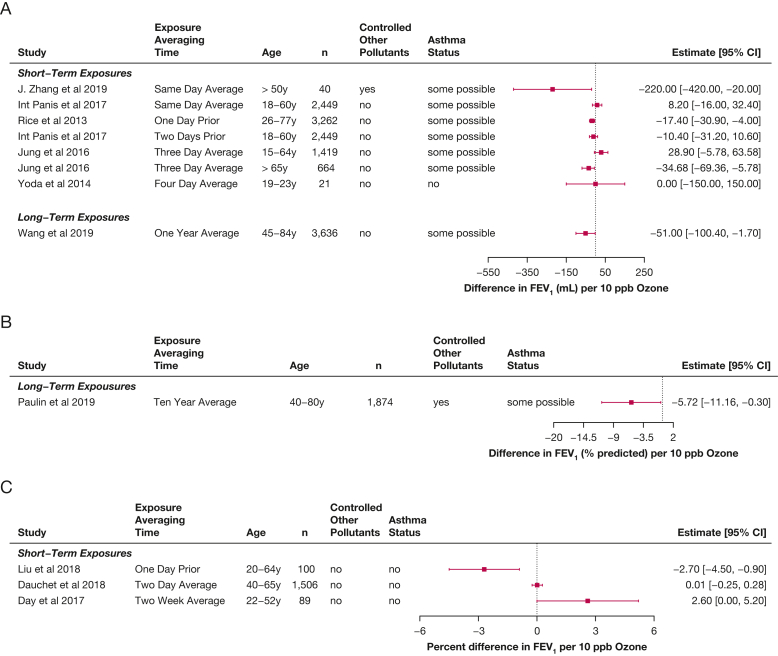
A-C, Forest plots showing estimates of differences in FEV_1_ associated with increases of 10 ppb ozone in adults, from studies published from 2013 through 2020 within the studies that reported differences in milliliters (A), within the studies that reported differences in percent predicted (B), and in studies that reported multiplicative changes (C), in percent differences.

Multiple new epidemiologic studies of ozone exposures and lung function in adults were published during the review period. By design, these studies were conducted at real-life levels of exposure (mean ozone levels in these studies ranged from 1 to 42 ppb), with associated coexposures.^7^^,^^37^, ^38^, ^39^, ^40^, ^41^, ^42^, ^43^, ^44^ All of these studies involved either general population cohorts or cohorts of healthy adults. The observed associations were of small magnitude or null, with decreases in FEV_1_ usually associated with longer durations of exposure (eg, multiple days) and in the elderly (Fig 5). The potential for bias may exist in these studies because they largely do not account for exposures to copollutants. The one study that did adjust for coexposures also included personal ozone monitoring^37^ and found a decrease of 220 mL associated with increases of 10 ppb in ozone, when adjusted for fine particles (PM_2.5_) (95% CI, –420 to –20 mL), suggesting that the other studies may have underestimated the ozone effect. In the only study that evaluated multiple age groups of adults,^7^ a decrease in FEV_1_ was associated with ozone exposure in the elderly (in those older than 65 years, –35 mL [95% CI, –69 to –6 mL]), but not in other adults (15-64 years of age, 29 mL [95% CI, –6 to 64 mL]). This is at odds with older published literature in which smaller effects are seen in the elderly compared with younger adults.

**Figure 6:**
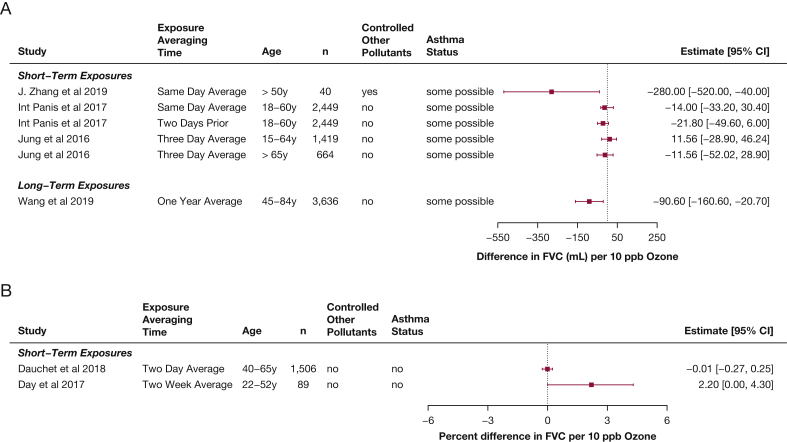
A-B, Forest plots showing estimates of differences in FVC associated with increases of 10 ppb ozone in adults, from studies published from 2013 through 2020 within the studies that reported differences in milliliters (A) and in studies that reported multiplicative changes (B), in percent differences.

Regarding FVC, similar patterns were found as seen as for FEV_1_, although the effects were generally smaller (Fig 6),^7^^,^^38^^,^^40^ with larger decreases seen in the elderly.^7^^,^^37^ Two of the studies in younger adults found increased FVC associated with ozone exposure,^7^^,^^41^ and a study that assessed indoor home ozone found an increase in FEV_1_ to FVC ratio associated with ozone exposure.^43^

Two studies that presented Results only graphically were left off the forest plots,^45^^,^^46^ but both seemed to show nonsignificant decreases in FEV_1_ and FVC associated with same-day ozone exposure. Additionally, one study^47^ used only repeated measurements of PEF to assess lung function in adults and found decreases associated with increased ozone exposure (–0.66 L/m; 95% CI, –1.21 to –0.12 per 10 ppb ozone).

A few studies looked at specific subpopulations of adults, including those with pre-existing illness. Twelve-month ozone exposure was associated with an increased FEV_1_ of 3.95% predicted and FVC of 2.15% predicted in lung transplant recipients,^48^ which may be partially the result of strong inverse correlations between ozone and other air pollutants in this study. Ozone exposure was associated with a decrease of 0.41% predicted for FVC associated with long-term ozone in idiopathic pulmonary fibrosis patients.^49^ In dairy workers, a doubling of low-level ozone exposure was not associated with differences in FEV_1_ or FVC.^50^ Two different studies considered lung function changes in adults with COPD in association with ozone. In patients with COPD with a history of lung volume reduction surgery, same-day ozone exposure was associated with an increase in % predicted FEV_1_ (4.94% predicted; 95% CI, –7.92% to 17.80% predicted) and a decrease in % predicted FVC (–14.93% predicted; 95% CI, –1.03% to 29.33% predicted).^51^ In a broader cohort of patients with COPD, ozone exposure lagged 3 to 5 days was associated with decreases in FEV_1_, with the largest effect at the 5-day lag (–5.92% change in mean FEV_1_, measured in liters; 95% CI, –11.20% to –0.64%).^52^

The epidemiologic evidence in the review interval suggests a more harmful effect in older adults of short-term ozone exposure. This is somewhat contradictory to prior controlled exposure evidence that suggests a greater effect of ozone exposure on lung function in younger adults.

### Long-Term Ozone and Lung Function in Children and Adolescents

An increased number of studies evaluating the relationship between long-term ozone exposure and lung function in children was published during the review period (Fig 2). Thirteen new studies show decreases in lung function associated with long-term exposures (mean range of ozone levels in these studies, 3-44 ppb). Among the seven studies that report Results related to ozone averaged over 2 years or more, a fairly consistent pattern of decreases in FEV_1_ is reported (+15 mL to –71 mL, –9% predicted, or a 1 % decrease all per 10 ppb ozone).^10^^,^^12^^,^^13^^,^^18^^,^^31^^,^^33^^,^^53^, ^54^, ^55^ Among those studies that assessed ozone exposures of 1 month to 1 year, a relationship with FEV_1_ in children does not seem to exist.^11^^,^^14^^,^^53^^,^^56^^,^^57^ Only four of these ozone-lung function estimates included copollutant control, suggesting that the effect could be underestimated.^10^^,^^13^^,^^14^^,^^18^

Two studies evaluated lung function growth during adolescence: those reported in Hwang et al^10^ and the Children’s Health Study (in both an Health Effects Institute report and a published article).^55^^,^^58^ The Hwang et al study showed a large decrease in lung function growth associated with exposure to ozone over the prior 2 years for both FEV_1_ (–41.4 mL; 95% CI, –67.54 to –15.31 mL) and FVC (–45.41 mL; 95%, –71.39 to –19.42 mL).^10^ In the study by Gauderman et al,^55^ a nonsignificant trend toward a decrease in lung function growth from 11 to 15 years was found, although the association of the ozone exposure with 15-year data alone suggested a trend toward an increase in lung function measures. Taken together, these suggest that cross-sectional analyses may mask some of the effects of ozone exposure, which are particularly strong when evaluating growth in FEV_1_.

Nine of the 13 studies that reported results for long-term exposure to ozone and FEV_1_ also reported results for FVC (Fig 3).^10^, ^11^, ^12^, ^13^^,^^18^^,^^33^^,^^54^^,^^56^^,^^57^ Unlike for FEV_1_, the results for FVC overall do not seem to suggest a clear relationship between long-term ozone exposure and FVC, although a few studies have found a relationship.^13^^,^^14^ The two studies that assessed lung function growth in adolescence^10^^,^^55^ report conflicting results, with a clear decrement in FVC growth associated with ozone exposure in the Hwang et al study,^10^ but a null finding in the Gauderman et al study.^55^ Two studies additionally reported PEF results associated with long-term exposure to ozone (Fig 4) and also found significant decreases.^18^^,^^33^

Assessing the overall pattern, growth of FEV_1_ may be particularly susceptible to ozone effects; patterns for the relationship between FVC and ozone were less clear. Larger decrements in % predicted FEV_1_ seem to be associated with longer-term increases in exposure to ozone in children (eg, multiple years) compared with shorter-term increases, including in the studies that assessed children using multiple exposure averages.^11^^,^^13^^,^^53^

### Long-Term Ozone and Lung Function in Adults

Only three new reports of long-term ozone exposure and lung function in adults were published during the review period. Paulin et al^59^ found a decrease in FEV_1_ associated with 10-year ozone exposure in current or former smokers (–2.10% predicted FEV_1_; 95% CI, –4.10% to –0.11% predicted). In a large cohort study, decreases in baseline lung function associated with 1-year average ozone exposure were reported (FEV_1_, –51.0 mL [95% CI, –100.4 to 1.7 mL]; and FVC, –90.6 mL [95% CI, –160.6 to –20.7 mL]) and in the change in lung function associated with interval ozone exposure (median interval, 10 years; FEV_1_, –53.2 mL [95% CI –104.3 to –2.0 mL]; and FVC, –104.8 mL [95% CI, –192.9 to –16.7 mL]).^60^ In patients with COPD with a history of lung volume reduction surgery, 6- to 12-month ozone exposure was associated with a decrease in both % predicted FEV_1_ (–0.227% predicted; 95% CI, –0.539% to 0.085% predicted) and FVC (–0.499% predicted; 95% CI, –1.03% to 0.030% predicted).^51^

## Discussion

A substantial body of research on the respiratory effects of ozone was published during the 8-year period of this review. Multiple studies involved average ozone levels at or less than the current US EPA standard (70 ppb over an 8-h averaging time), with the exception of the article exploring genotypes,^29^ which reported mean levels up to 97 ppb. Although the studies in adults do not show a clear relationship between low-level ozone exposure and lung function, those in children suggest that even at low levels, well below the current EPA standard, children’s lung function may be affected adversely by ozone exposure, particularly over longer periods. Thus, the current EPA NAAQS may not be adequately protective of children’s respiratory health.

However, further research is needed, particularly regarding changes in lung function with long-term ozone exposure. Most of the reported research has come from North America and Europe, whereas large areas (eg, East Asia, the Sahara) that have substantial ozone exposure are underrepresented. Also, a need exists for further elucidation of particularly susceptible subgroups regarding long-term ozone exposures.

We summarized the relevant epidemiologic studies, but some limitations to this literature exist. First, few of the studies account for copollutant exposures. In addition, different investigators have used different metrics to summarize ozone exposure (24-h averages, highest 8-h average concentrations, maximum daily values), and regulatory bodies in different countries use volume (ppb) or mass (μg/m^3^) metrics. The current EPA NAAQS^61^ is for a maximum 8-h daily average; the fourth highest 8-h average value over a 3-year period is the actual value that must be less than 70 ppb (0.070 ppm). The ozone target value for the European Union is a maximum daily 8-h average of 120 μg/m^3^ with 25 exceptions allowed over a 3-year period.^62^ These differing standards mean that research from different regions target different metrics, increasing the difficulty of making new policy decisions based on the literature.

Much of the modeling has been carried out assuming a linear relationship between ozone concentration and lung function effects, and all but one of the studies reviewed here relied on linear models. Yet, investigators who have attempted to study ozone exposure-response curves for lung function have found a suggestion of nonlinearity^59^ similar to the typically nonlinear relationships seen for other outcomes (eg, mortality).^63^^,^^64^ Moreover, a linear model assumes that it is possible to have the exposure reach zero, which is a nonsensical assumption when the exposure is ambient ozone.

## Interpretation

In summary, adverse effects on lung function from relatively low-level exposure to ozone are likely, especially for children and possibly the elderly. Mounting evidence suggests that children’s lung function may be affected by long-term ozone exposure, particularly when considering lung function growth. Therefore, the current US EPA ozone NAAQS may not provide a sufficient margin of safety to protect public health.

**Take-home Points**

**Study Question:** What has been learned about the association between ozone exposures and lung function from studies published from 2013 through 2020?

**Results:** Multiple new studies have demonstrated associations between low-level ozone exposure and decreases in children’s lung function, both for short- and long-term averages; the new literature in adults is less clear.

**Interpretation:** Evidence is accumulating for health effects of ozone of less than the current 70 parts per billion ambient air quality standard, particularly in children.

## Supplementary Data

e-Online Data

e-Online Data

## References

[1] Balmes J.R., Holm S.M., Broaddus V.C., Ernst J.D., King T.E. (2022). Murray & Nadel’s Textbook of Respiratory Medicine.

[2] United States Environmental Protection Agency Integrated Science Assessment (ISA) for ozone and related photochemical oxidants (final report, Apr 2020). Environmental Protection Agency website. https://cfpub.epa.gov/ncea/isa/recordisplay.cfm?deid=348522.

[3] Holm S.M., Balmes J.R., Roy A. Human health effects of ozone: the state of evidence since EPA’s last Integrated Science Assessment. 2018. Environmental Defense Fund website. https://www.edf.org/sites/default/files/content/Ozone_Summary_Report.pdf.

[4] Sheehan M.C., Lam J., Navas-Acien A., Chang H.H. (2016). Ambient air pollution epidemiology systematic review and meta-analysis: a review of reporting and methods practice. Environ Int.

[5] Cochrane Scientific Committee Review of the development of the risk of bias tool for nonrandomised studies for interventions—ROBINS-I. 2017 [cited]. Cochrane website. https://methods.cochrane.org/sites/default/files/public/uploads/scientific_committee_statement_report_robins_i_fin.pdf.

[6] Sterne J.A., Hernán M.A., Reeves B.C. (2016). ROBINS-I: a tool for assessing risk of bias in non-randomised studies of interventions. BMJ.

[7] Jung S.-W., Lee K., Cho Y.-S. (2016). Association by spatial interpolation between ozone levels and lung function of residents at an industrial complex in South Korea. Int J Environ Res Public Health.

[8] Karakatsani A., Samoli E., Rodopoulou S. (2017). Weekly personal ozone exposure and respiratory health in a panel of Greek schoolchildren. Environ Health Perspect.

[9] Yoda Y., Takagi H., Wakamatsu J. (2017). Acute effects of air pollutants on pulmonary function among students: a panel study in an isolated island. Environ Health Prev Med.

[10] Hwang B.-F., Chen Y.-H., Lin Y.-T., Wu X.-T., Leo Lee Y. (2015). Relationship between exposure to fine particulates and ozone and reduced lung function in children. Environ Res.

[11] Ierodiakonou D., Zanobetti A., Coull B.A. (2016). Ambient air pollution, lung function, and airway responsiveness in asthmatic children. J Allergy Clin Immunol.

[12] Neophytou A.M., White M.J., Oh S.S. (2016). Air pollution and lung function in minority youth with asthma in the GALA II (Genes-Environments and Admixture in Latino Americans) and SAGE II (Study of African Americans, Asthma, Genes, and Environments). Studies. Am J Respir Crit Care Med.

[13] Tsui H.-C., Chen C.-H., Wu Y.-H., Chiang H.-C., Chen B.-Y., Guo Y.L. (2018). Lifetime exposure to particulate air pollutants is negatively associated with lung function in non-asthmatic children. Environ Pollut.

[14] Chen C.-H., Chan C.-C., Chen B.-Y., Cheng T.-J., Leon Guo Y. (2015). Effects of particulate air pollution and ozone on lung function in non-asthmatic children. Environ Res.

[15] Dales R.E., Cakmak S. (2016). Does mental health status influence susceptibility to the physiologic effects of air pollution? A population based study of Canadian children. PLoS One.

[16] Barbone F., Catelan D., Pistelli R. (2019). A Panel Study on Lung Function and Bronchial Inflammation among Children Exposed to Ambient S02 from an Oil Refinery. Int J Environ Res Public Health.

[17] Li Y.-R., Feng L.-T., Chen B.-Y. (2016). Association of urban particle numbers and sources with lung function among children with asthma or allergies. Science of the Total Environment.

[18] Dimakopoulou K., Douros J., Samoli E. (2020). Long-term exposure to ozone and children’s respiratory health: Results from the RESPOZE study. Environ Res.

[19] Zhang J., Feng L., Hou C., Gu Q. (2020). How the constituents of fine particulate matter and ozone affect the lung function of children in Tianjin, China. Environ Geochem Health.

[20] Huang J., Song Y., Chu M. (2019). Cardiorespiratory responses to low-level ozone exposure: the inDoor Ozone Study in childrEn (DOSE). Environ Int.

[21] Hernandez M.L., Dhingra R., Burbank A.J. (2018). Low-level ozone has both respiratory and systemic effects in African American adolescents with asthma despite asthma controller therapy. J Allergy Clin Immunol.

[22] National Heart, Lung, and Blood Institute (2007).

[23] Kurai J., Noma H., Sano H., Iwata K., Tohda Y., Watanabe M. (2018). Association of short-term ozone exposure with pulmonary function and respiratory symptoms in schoolchildren: a panel study in a western Japanese city. J Med Invest.

[24] Samoli E., Dimakopoulou K., Evangelopoulos D. (2017). Is daily exposure to ozone associated with respiratory morbidity and lung function in a representative sample of schoolchildren? Results from a panel study in Greece. J Expo Sci Environ Epidemiol.

[25] Adman M.A., Hashim J.H., Manaf M.R.A., Norback D. (2020). Associations between air pollutants and peak expiratory flow and fractional exhaled nitric oxide in students. Int J Tuberc Lung Dis.

[26] Amadeo B., Robert C., Rondeau V. (2015). Impact of close-proximity air pollution on lung function in schoolchildren in the French West Indies. BMC Public Health.

[27] Hasunuma H., Yamazaki S., Tamura K. (2018). Association between daily ambient air pollution and respiratory symptoms in children with asthma and healthy children in western Japan. J Asthma.

[28] Chen B.-Y., Chen C.-H., Chuang Y.-C. (2016). Schoolchildren’s antioxidation genotypes are susceptible factors for reduced lung function and airway inflammation caused by air pollution. Environ Res.

[29] Moreno-Macías H., Dockery D.W., Schwartz J. (2013). Ozone exposure, vitamin C intake, and genetic susceptibility of asthmatic children in Mexico City: a cohort study. Respir Res.

[30] Altuğ H., Gaga E.O., Döğeroğlu T. (2013). Effects of air pollution on lung function and symptoms of asthma, rhinitis and eczema in primary school children. Environ Sci Pollut Res.

[31] Zeng X.-W., Vivian E., Mohammed K.A. (2016). Long-term ambient air pollution and lung function impairment in Chinese children from a high air pollution range area: the Seven Northeastern Cities (SNEC) study. Atmos Environ.

[32] Zhang C., Guo Y., Xiao X. (2019). Association of breastfeeding and air pollution exposure with lung function in Chinese children. JAMA Netw Open.

[33] Xing X., Hu L., Guo Y. (2020). Interactions between ambient air pollution and obesity on lung function in children: the Seven Northeastern Chinese Cities (SNEC) Study. Science of the Total Environment.

[34] Benka-Coker W., Hoskovec L., Severson R., Balmes J., Wilson A., Magzamen S. (2020). The joint effect of ambient air pollution and agricultural pesticide exposures on lung function among children with asthma. Environ Res.

[35] Fernández-Plata R., Rojas-Martínez R., Martínez-Briseño D., García-Sancho C., Pérez-Padilla R. (2016). Effect of passive smoking on the growth of pulmonary function and respiratory symptoms in schoolchildren. Revista de Investigacion Clinica.

[36] Pasalic E., Hayat M., Greenwald R. (2016). Air pollution, physical activity, and markers of acute airway oxidative stress and inflammation in adolescents. J Ga Public Health Assoc.

[37] Zhang J., Sun H., Chen Q., Gu J., Ding Z., Xu Y. (2019). Effects of individual ozone exposure on lung function in the elderly: a cross-sectional study in China. Environ Sci Pollut Res.

[38] Int Panis L., Provost E.B., Cox B. (2017). Short-term air pollution exposure decreases lung function: a repeated measures study in healthy adults. Environ Health.

[39] Yoda Y., Otani N., Sakurai S., Shima M. (2014). Acute effects of summer air pollution on pulmonary function and airway inflammation in healthy young women. J Epidemiol.

[40] Dauchet L., Hulo S., Cherot-Kornobis N. (2018). Short-term exposure to air pollution: associations with lung function and inflammatory markers in non-smoking, healthy adults. Environ Int.

[41] Day D.B., Xiang J., Mo J. (2017). Association of ozone exposure with cardiorespiratory pathophysiologic mechanisms in healthy adults. JAMA Intern Med.

[42] Rice M.B., Ljungman P.L., Wilker E.H. (2013). Short-term exposure to air pollution and lung function in the Framingham Heart Study. Am J Respir Crit Care Med.

[43] Bräuner E.V., Karottki D.G., Frederiksen M. (2016). Residential ozone and lung function in the elderly. Indoor Built Environ.

[44] Liu J.-Y., Hsiao T.-C., Lee K.-Y., Chuang H.-C., Cheng T.-J., Chuang K.-J. (2018). Association of ultrafine particles with cardiopulmonary health among adult subjects in the urban areas of northern Taiwan. Science of the Total Environment.

[45] Zhou Y., Liu Y., Song Y. (2016). Short-term effects of outdoor air pollution on lung function among female non-smokers in China. Sci Rep.

[46] Lepeule J., Bind M.-A.C., Baccarelli A.A. (2014). Epigenetic influences on associations between air pollutants and lung function in elderly men: the Normative Aging Study. Environ Health Perspect.

[47] Chang L.-T., Hong G.-B., Weng S.-P. (2019). Indoor ozone levels, houseplants and peak expiratory flow rates among healthy adults in Taipei, Taiwan. Environ Int.

[48] Benmerad M., Slama R., Botturi K. (2017). Chronic effects of air pollution on lung function after lung transplantation in the Systems Prediction of Chronic Lung Allograft Dysfunction (SysCLAD) study. Eur Respir J.

[49] Johannson K.A., Vittinghoff E., Morisset J. (2018). Air pollution exposure is associated with lower lung function, but not changes in lung function, in patients with idiopathic pulmonary fibrosis. Chest.

[50] Martenies S.E., Schaeffer J.W., Erlandson G. (2020). Associations between bioaerosol exposures and lung function changes among dairy workers in Colorado. J Occup Environ Med.

[51] Kariisa M., Foraker R., Pennell M. (2015). Short- and long-term effects of ambient ozone and fine particulate matter on the respiratory health of chronic obstructive pulmonary disease subjects. Arch Environ Occup Health.

[52] Li H., Wu S., Pan L. (2018). Short-term effects of various ozone metrics on cardiopulmonary function in chronic obstructive pulmonary disease patients: Results from a panel study in Beijing, China. Environmental Pollution.

[53] Usemann J., Decrue F., Korten I. (2019). Exposure to moderate air pollution and associations with lung function at school-age: a birth cohort study. Environ Int.

[54] Urman R., McConnell R., Islam T. (2014). Associations of children’s lung function with ambient air pollution: joint effects of regional and near-roadway pollutants. Thorax.

[55] Gauderman W.J., Urman R., Avol E. (2015). Association of improved air quality with lung development in children. N Engl J Med.

[56] Fuertes E., Bracher J., Flexeder C. (2015). Long-term air pollution exposure and lung function in 15 year-old adolescents living in an urban and rural area in Germany: the GINIplus and LISAplus cohorts. Int J Hygiene Environ Health.

[57] Barone-Adesi F., Dent J.E., Dajnak D. (2015). Long-term exposure to primary traffic pollutants and lung function in children: cross-sectional study and meta-analysis. PLoS One.

[58] Gilliland F, Avol E, McConnell R, et al. Effects of policy-driven air quality improvements on children’s respiratory health. Health Effects Institute Research report. Report number 190, January 2017. PMC7266378 31898879

[59] Paulin L.M., Gassett A.J., Alexis N.E. (2020). Association of long-term ambient ozone exposure with respiratory morbidity in smokers. JAMA Intern Med.

[60] Wang M., Aaron C.P., Madrigano J. (2019). Association between long-term exposure to ambient air pollution and change in quantitatively assessed emphysema and lung function. JAMA.

[61] United States Environmental Protection Agency Ozone national ambient air quality standards (NAAQS). 2020. Environmental Protection Agency website. https://www.epa.gov/ground-level-ozone-pollution/ozone-national-ambient-air-quality-standards-naaqs.

[62] European Commission Air quality standards. European Commission website. https://ec.europa.eu/environment/air/quality/standards.htm.

[63] Bae S., Lim Y.-H., Kashima S. (2015). Non-linear concentration-response relationships between ambient ozone and daily mortality. PLoS One.

[64] Collart P., Dramaix M., Levêque A., Mercier G., Coppieters Y. (2018). Concentration-response curve and cumulative effects between ozone and daily mortality: an analysis in Wallonia. Belgium. Int J Environ Health Res.

