# Supplementary material for: Systematic Review of Ozone Effects on Human Lung Function, 2013 Through 2020
Source: Chest. 2021 Aug 10;161(1):190–201. doi: 10.1016/j.chest.2021.07.2170 (PMC8783034; doi:10.1016/j.chest.2021.07.2170)
Supplement: e-Online Data [file mmc1.pdf]

CHEST Online Supplement

Systematic Review of Ozone Effects on Human Lung Function, 2013 Through 2020

Stephanie M. Holm, MD, PhD, MPH; and John R. Balmes, MD

CHEST 2022; 161(1):190-201

Online supplements are not copyedited prior to posting and the author(s) take full responsibility for the accuracy of all data.

© 2021 AMERICAN COLLEGE OF CHEST PHYSICIANS. Reproduction of this article is prohibited without written permission from the American College of Chest Physicians. See online for more details. **DOI:** 10.1016/j.chest.2021.07.2170

e-Appendix 1. Respiratory: Lung Function

Paper	Study design	Population	Ozone Exposure	Outcome	Notes
**Lung Function**					
Adman et al 2020[1]	Cross sectional assessment in Malaysia, 2014	487 students, recruited from 4 secondary schools in two cities (all 14yo students invited to participate)	From one continuous air quality monitoring station in each city	In single pollutant models, 10 ppb of one day ozone associated with -13.2 l/min of PEF (-22.67, -3.74), two day with -19.12 (-31.24, -7.00), three days with -27.72 (-42.76, -12.68) and 7 days with -30.16 (-47.59, -12.73). Temperature and humidity were also associated with PEF.	Median ozone levels approx. 20 ppb with maximum one day averages of 29 ppb.
Altug et al 2013[2]	Cohort study in Eskisehir, Turkey	1880 students aged 9-13 years followed Jan 2008-Mar 2009	Passive samplers were set up for two weeks at each school where students were located	Increment of 10 μg/m^3 ozone associated with increased odds of impaired lung function in girls during the summer season only, OR 1.11(1.03,1.19). No association found with wheeze, rhinoconjunctivitis or itchy rash. Each student had lung function assessed 2x.	Ozone averages ranged 24-87 μg/m^3 in the winter and 72-110 μg/m^3 in the summer
Amadeo et al 2015[3]	Cross-sectional study at 30 elementary schools in the French West Indies	1463 children (age 8-13) who completed questionnaire and performed lung function; 277 kids with asthma	Close proximity ozone measured in each playground and outside at each playground for two weeks prior to assessment. Background ozone estimated for monitoring data for a subset of 7 schools.	1 μg/m^3 increase in close-proximity outdoor ozone over two weeks associated with -0.32 L/min (decrease) in peak expiratory flow (-0.61, -0.03). Indoor ozone not significantly associated with PEF. Background outdoor ozone also not associated with PEF. Though not significant, there may be a greater effect of ozone on PEF in asthmatic children -1.88 L/m (-4.5, 0.74).	Mean two-week indoor ozone was 49.5 μg/m^3 and outdoor ozone was 55.3 μg/m^3. Mean estimated background ozone was 54.1 μg/m^3
Barbone et al 2019[4]	Cohort study in Sarroch, Italy	233 children (age 8-14) with monthly visits for 5 months (subgroup of 54 with asthma or wheezing history did weekly visits). Enrolled all children in the local schools.	Hourly pollutant levels collected from three community monitoring stations.	A 10 μg/m^3 increase in ozone averaged over the current and prior two days associated with a percent variation of -1.31 (-1.71, 0.90) in FEV1 among the community cohort and -1.02 (-1.6, -0.44) among the children with a wheezing history.	Note that this town was selected based on its proximity to a major oil refinery. Mean ozone averaged over the prior two days was 62.8 μg/m^3

Online supplements are not copyedited prior to posting and the author(s) take full responsibility for the accuracy of all data.

Barone-Adesi 2015[5]	Cross-sectional study in London, England	4884 9-10-year-old children who participated in the Child Heart and Health study in England	Annual average pollutant levels modeled throughout London (20m x20m resolution)	Ozone not significantly associated with FEV1 or FVC. Per 2.9 microg/m3 increase in ozone, FEV1 increased 6 mL (-7, 20) and FVC 14 mL (-2,30).	Mean ozone 37.4 μg/m³, with IQR 2.9 μg/m³<br><br>Note that only 2/3 of those initially invited to participate did so.
Benka-Coker et al 2020[6]	Cross-sectional analysis of a cohort in the San Joaquin Valley of California	153 children with asthma (6-11y)	From the EPA monitor closest to the participant's home address. Daily averages were used to generate 3 month averages.	Analysis designed to look at mixtures in exposures including air pollutants and pesticides. In the Bayesian analysis, ozone less likely to be included in the mixture analysis compared to fine PM or NO2.<br><br>In the Bayesian model- slight, approximately linear decreases in FEV1, FVC and FEF25-75 associated with increases in ozone.<br><br>In linear models adjusted for other pollutants, per 12.4 ppb ozone, FEV1 decreased by -0.19 Z scores (-0.49, 0.11), FVC decreased by -0.22 Z scores(-0.52, 0.07) and FEF25-75 increased by 0.03 Z scores (-0.30, 0.35).	Mean ozone 35.8 ppb, range 12.3-58.2 ppb. IQR 16.4
Benmerad et al 2017[7]	Longitudinal cohort study	520 lung transplant recipients with spirometry every 6 months for up to four years	Modeled ozone at residential address for the 12 months prior to spirometry	10 μg/m³ increase in 12-month prior ozone associated with increased In FEV1 of 3.95 L, and FVC of 2.15 L..	Median ozone 52.5 μg/m³
Bräuner et al 2016[8]	Cohort of volunteers in Copenhagen, Denmark	51 non-smokers over age 50	Passive samplers in bedroom and living room, outdoor concentrations from a single outdooor community monitor	A 1 ppb increase in 2 week average ozone was associated with a 7.03 % increase in FEV1/FVC ratio (-0.29, 15.0). Among those with asthma or obstructive lung disease (n=7), there was a 38% decrease: -38.2% (-59.7, -3.00).<br><br>Note that the results present data for the ratio only, and not for FEV1 nor FVC individually (and thus not included in figures).	This is a re-analysis of a filtration intervention where the filtration did not affect the ozone concentrations. Mean indoor ozone was 1.33 ppb, mean outdoor ozone was 27.0 ppb. (Max measured was 41.4 ppb)

Online supplements are not copyedited prior to posting and the author(s) take full responsibility for the accuracy of all data.

Chang et al 2019[9]	Cohort in Taiwan, followed with monthly visits for one year.	60 non-smoking homemakers aged 30-45, half of whom had houseplants	Monitored indoors at home for the 24 hours prior to the assessment. Outdoor data from the nearest station of the Taiwan EPA.	An IQR increase in ozone among all subjects was associated with a 1.32 L/m decrease (-2.41, -0.23) in PEF. This seems to largely be driven by strong associations with ozone in the summer among subjects who have houseplants (-6.88, 95% CI -9.52, -4.24), which are those with the highest exposure.	Ozone IQR was 20.4 ppb. Mean indoor ozone was 47.5 ppb for those with houseplants and 28.4 for those without (38 ppb overall). Mean outdoor ozone was 34.1 ppb.
Chen et al 2015[10]	National school-based study in Taiwan	1494 children without asthma, ages 6-15	From air monitoring system data in each community, within 1km of the 44 schools where children were tested. Summarized as both lag 1 and lag2 day, as well as with a 2 month average.	Per IQR, for lag 1 day ozone (adjusted for PM2.5), FVC decreased by -27.1 mL (SE 29) and FEV1 by -36 mL (SE 30.3). The ratio decreased -0.81 percent (SE 0.311).<br><br>Per IQR, for 2 month average ozone (adjusted for PM2.5), FVC decreased by -78.4 mL (SE 35.4) and FEV1 by -83.2 mL (SE 27.8).<br><br>In an analysis split by age (6-10 versus 11-15), the effects appear possibly larger in the older children, though the interaction term did not reach significance.	Mean lag 1 day ozone 28.95 ppb, IQR 19.35.<br><br>Mean 2 month ozone 34.45 ppb, IQR 6.67
Chen et al 2016 (Envronmental Research)[11]	Cohort study in Taipei, Taiwan	97 schoolchildren followed for 2 years, with schools within 2.5 km of the environmental monitoring site. Had lung function monthly.	From the Taiwanese Environmental Protection Agency monitoring site. 8-hour averages were used for ozone, and analyses were done with one day lags	FEF 25, FEF50, FEF75 and FEF25-75 were decreased with an IQR increase in ozone, those decreases were more prominent in participants with the SOD2 V16A Val/Ala or Ala/Ala genotypes compared to those with Val/Val. That difference for the genotypes was significant only for FEF25 where those with val/val did not decrease significantly and those with val/ala or ala/ala decreased 0.43 L/s (-0.58, -0.28).	IQR of ozone 12.9 ppb, mean ~ 30 ppb
Dales and Cakmak 2016[12]	Cross-sectional study, from a larger population representative cohort	1883 children, ages 6-17 years from the Canadian Health Measures population survey (1770 without a mood disorder)	From the national air pollution monitoring site closest to the home address on the day of testing	For an IQR increase in ozone, among children without mood disorders, there was a small % decrease in percent predicted FEV1 (-1.08, 95% CI -2.08 to -0.07), FVC (-0.52, 95% CI -1.5 to 0.47) and FEV1/FVC ratio (-0.65, 95% CI -1.23, -0.07).<br><br>However, in children with mood disorders there was a much larger % decrease in both FEV1 and FVC associated with ozone (-6.18, 95% CI -10.63, -1.73; -7.62, 95% -12.31, -2.92), but no difference in the ratio.<br><br>Among children who were not happy, there was a significant % decrease in FEV1/FVC ratio (-2.15, 95% CI -3.62 to -0.68), but only small (non-significant) decreases in FEV1 and FVC.	Mean 8 hour ozone max level was 29.50 ppb (IQR 17 ppb).<br><br>Note that children with some symptoms of emotional distress had higher mean ozone (31.4) than those with no symptoms.

Online supplements are not copyedited prior to posting and the author(s) take full responsibility for the accuracy of all data.

				Among children who had one or more adverse emotional symptoms, there were % decreases in all three that were larger than those without the symptoms (though the finding is significant only for the ratio): FEV1 -5.97 (-10.57, -1.37), FVC -1.78 (-6.19, 2.83) and FEV1/FVC -5.91 (-9.91, -1.91).	
Dauchet et al 2018[13]	Cross-sectional study, from a larger population representative cohort	1506 non-smoking adults (age 40-65) with no respiratory disease	From air pollution monitoring sites in the two cities, using only data from closely correlated monitors to capture general ambient pollution in the cities.	Per 10 μg/m³ same day and day prior ozone, there was effectively no association with spirometry. FEV1 in L had a 0.02% change (95% CI -0.50, 0.54), FVC in L had a -0.02% change (95% CI -0.53, 0.50) and FEV1/FVC ratio had a 0.02% change (95% CI -0.16, 0.20).<br><br>Note that participants who had airway obstruction on spirometry measurements were excluded from the study	Median ozone was 59.5 μg/m³ in Lille (IQR 32.7) and in Dunkirk was 57.8 (IQR 26.7)
Day et al 2017[14]	Longitudinal cohort at a company campus where people both live and work in China	89 healthy white-collar workers (22-52y), each with 4 visits over a 9 week period, during which a particle filtration intervention was being assesses	Outdoor measurements from a local monitoring site combined with indoor measurements in the offices and dormitories. Participants were assigned personal exposures using time-weighted averages.	For a 10 ppb increase in 2 week ozone exposure there was an increase in FEV1 in L of 2.6% (95% CI 0.02, 5.2) and in FVC 2.2% (95% CI 0.04, 4.3).<br><br>Effects for 24h averages appear very similar from plots, but no point estimates are reported. In two pollutant models, again effects appear similar but no point estimates reported.<br><br>Multi-pollutant models were similar (in the supplement) but numeric results were only presented for the single-pollutant models, so that was what was included in graphs.	Median 24 hour ozone exposure was 4.59 ppb (IQR 18.00).<br><br>Median 2 week ozone exposure was 6.95 (IQR 8.82)
Dimakopoulou et al 2020[15]	Cohort followed for 5 weeks each during the school year	186 10-11 year olds in Athens and Thessalonaki, Greece, attending schools in either high or low ozone areas (within 2 km of a monitoring station)	Data used from both fixed monitoring stations (6 in Athens, 3 in Thessaloniki) using the station closest to the participant's home and from dispersion models calculated at the home address. Personal measurements were used to adjust long term measurements for time spent	Per 10 μg/m³ of ozone, (adjusted for PM10 and roadway proximity) FVC decreased -0.019 L (95% CI -0.031, -0.007) and FEV1 decreased -0.014 (95% CI -0.023, -0.004). PEF not substantially different, -0.013 L/sec (-0.057, 0.031).<br><br>Results similar whether measured with fixed stations or dispersion models.<br><br>For lung function growth over a 5 week period, per 10 μg/m³ of two-year average ozone (adjusted for roadway proximity and PM10), there	Using the fixed site monitors adjusted for personal monitoring, low ozone group had 2 year exposures 5.1-68.8 μg/m³ and high ozone group ranged from 27.2-236.8 μg/m³.

Online supplements are not copyedited prior to posting and the author(s) take full responsibility for the accuracy of all data.

			indoor. Long-term measurements were two year averages over 2013-2014	was a decrease of -0.009 percent (-0.015, -0.002) in FVC growth and -0.007 percent (-0.013, -0.001) in FEV1 growth.	
Fernandez-Plata et al 2016[16]	Longitudinal cohort study	3187 participants followed for up to six years in Mexico, whose schools were within 2 km of an air monitoring station. Visits were every 6 months	Daily max 8-hour average of ozone from the monitor closest to the child's school, averaged over the 6 months prior to spirometry	Those in the highest quartile of ozone exposure had 1.04 odds ratio for respiratory infections compared to those in the lowest quartile (1.03,1.05). And again, comparing quartiles, those with higher ozone had a decrease in the Z-scores of FEV1 of 0.010 (-0.012, -0.009), decreased FVC of 0.007 (-0.008, -0.005) and decreased FEV1/FVC ratio 0.007 (-0.009, -0.006).	Note- ozone results had previously been reported in Rojas 2007
Fuertes et al 2015[17]	Cohort study, but with only one measurement of lung function used	2266 fifteen-year old's in Germany	Modeled ozone at addresses at time of participants birth, age ten and current	No relationship with long term (annual) ozone and lung function measures. IQR change in ozone long-term: 7.18 mL FEV1 (-18.88, 33.25) and 10.86 mL FVC (-17.65, 39.37). Short term ozone was controlled for in the long-term models, but was measured only at central sites and not estimated individually.	Long term median 44 $\mu g/m^3$and short term median 70 $\mu g/m^3$. IQR range of ozone was 5.8 $\mu g/m^3$
Gauderman et al 2015[18]<br><br>Gilliland et al 2017[19]	Combined longitudinal cohort of children in 3 different calendar periods in Southern California (1994-1997, 1997-2000, 2007-2010)	2120 adolescents, 1585 of them were followed from roughly age 11 through 15	Monitored at the community level using local monitoring stations over the 4 year exposure period.	Per 5.5 ppb decrease in ozone:<br>At age 11 had no effect on FEV1 (15.0 mL, 95% CI -38.5, 68.6) or FVC (7.6 mL, 95% CI -50.2, 65.4).<br><br>At age 15, again had no effect on FEV1 (8.3 mL, 95% CI -82.9, 99.6) or FVC (0.3 mL, 95% CI -126.0, 126.5).<br><br>Additionally, there was no effect of ozone on lung function growth from age 11 to 15, with FEV1 -6.7 (95% CI -51.0, 37.5) and FVC -7.3 (95% CI -79.3, 64.6).	Mean ozone levels ranged from 28.6 to 61.9 ppb in the various communities over the relevant time periods.
Hasunuma et al 2018[20]	Case-crossover study with repeated measurements.	138 healthy children attending schools in Fukuoka and 71 children with asthma recruited from local hospitals, followed for 3 months with twice daily peak flow.	Hourly photochemical oxidants (thought to be primarily ozone) measured from local monitoring stations using ultraviolet absorption.	10 ppb increase in oxidants associated with 0.26% decrease in peak expiratory flow (compared to subject's max PEF) lagged 3 days for children without asthma (-0.49, -0.03). Decreases on other days (lagged) not significant.<br>For children with asthma not on long term medications, there were significant decreases in peak expiratory flow with 10 ppb increase in oxidants lagged 2,3, 4, or 5 days. The largest decline was with lag 5, -0.51% (-0.89, -0.12).<br>For children with asthma on long term medications, changes with oxidant increases were not significant.	Mean oxidants exposure for healthy children 42.8 ppb, for children with asthma not on long-term medications 4.04 ppb, and for children with asthma on long-term medications 36.5 ppb

Online supplements are not copyedited prior to posting and the author(s) take full responsibility for the accuracy of all data.

Hernandez et al 2018[21]	Cohort in Raleigh, North Carolina	23 African American teens (12-17y) with persistent asthma	Used data from a central site monitor (all participants lived within 30 miles)	Per IQR of ozone, 5 day moving average associated with -2.6 percent predicted in FEV1 (-6.8, 1.0) and a decrease of -2.9 in FVC (-5.9 , 0.3). The largest effects were seen for same day averages.<br><br>(These are estimated from figure 1). These results are from single pollutant models though it is noted in the text that models controlling for PM2.5 gave similar results.	Average daily ozone means ranged 2-56 ppb, 8-hour max concentrations ranged 14.8-69.6 ppb. Ozone IQR 16.5 ppb
Huang et al 2019[22]	Cohort study in Beijing, China	46 healthy children, aged 11-14 years	Real-time concentrations measured in classrooms.	Per IQR increase in Ozone,<br>FEV1 measured in liters decreased -7.4% (-22.2, 10.3) with a 2 day lag. FVC results not given. PEF decreased -10.0% (-22.0%, 3.8%)<br><br>Effects were smaller at 0 and 1 day lags but numeric estimates not provided.	Mean indoor ozone was 8.7 ppb, IQR 10.9 ppb
Hwang et al 2015[23]	Two-year prospective cohort study in Taiwan	2941 children age 12 at baseline followed from 2007-2009, two visits total, two years apart	Annual averages for air pollutants calculated at residential addresses, for ozone, calculated from 8-hour concentrations.	1 ppb increase in average ozone associated with the following in terms of the lung function growth: a decrease of 41.43 mL FVC (15.31, 67.54), a decrease of 45.41 mL FEV1 (19.42,71.39) and decrease of 60.10 mL/s FEF_{25-75} (8.06, 112.14). When split by gender, the boys had larger decreases for all three measures when exposed to ozone compared to girls.	Mean 8-hour ozone 38.93 ppb, IQR 10.72 ppb
Ierodiakonou et al 2016[24]	Cohort study	1003 asthmatic children participating in a clinical trial. 13 lung function measurements over 4 years	Daily pollutant concentration modeled at the zip code level of home address, using data from the closest monitor	Ozone not significantly associated with FEV1 or FVC. Without bronchodilation, same day ozone associated with -0.02 % predicted FEV1 (-0.270, 0.24) and 0.09 % predicted FVC (-0.110, 0.30); 1-week ozone with -0.15 % predicted FEV1 (-0.530, 0.24) and 0.03 % predicted FVC (-0.270, 0.33); and 4 month ozone with -0.50 % predicted FEV1 (-1.170, 0.17) and -0.25 % predicted FVC (-0.780, 0.28).<br><br>IQR increase in long term ozone (4 month average) associated with decreased FEV1/FVC ratio after bronchodilation only -0.4 (-0.8, -0.1).	Median ozone 22 ppb. IQR ranges in ozone within subjects were 15 ppb (same day), 14 ppb (1 week) and 11 ppb (4 month)
Int Panis et al 2017[25]	Longitudinal administrative cohort study with annual lung function measurements	2449 workers enrolled in a worker health surveillance program in Northern Belgium	Daily measurements from a nearby monitoring station of the Flanders Environment Agency	None of the lung function measures were associated with ozone. Same day ozone, FVC -7.0 mL (-16.6, 15.2) and FEV1 4.1 mL (-8.0, 16.2). Ozone two days prior, FVC -10.9 mL (-24.8, 3.0) and FEV1 -5.2 mL (-15.6, 5.3).	Mean ozone 44 μg/m3

Online supplements are not copyedited prior to posting and the author(s) take full responsibility for the accuracy of all data.

Johannson et al 2018[26]	Longitudinal Prospective Cohort Study, 33 ± 9 weeks per participant	25 patients with Idiopathic pulmonary fibrosis	California Air Resources Board data used to estimate pollutants at each participants' home address	Mean FVC % predicted (averaged over entire study) associated with maximum ozone level -.41 (-0.81, -0.02), but no association with mean ozone level.	Notes that no O3 measurements exceeded the EPA standards
Jung et al 2016[27]	Cross-sectional study in Gwangyang Bay, South Korea	2283 participants	8-hour max ozone from 10 ambient air monitoring stations in the region. These were summarized for each subject in 4 different ways- averaging, nearest monitor, inverse weighting and kriging. (Kriging had lowest estimated mean squared error, so it is what we have reported).	For children ages 9-14, per IQR increase in ozone, FVC decreased by 0.09-0.14 liters depending on the method used to model ozone exposure and the lag (1day, 2d, 0-1, 1-2, 0-2).<br>For children ages 9-14, per IQR increase in ozone, FEV1 decreased by 0.06-0.12 liters depending on the method used to model ozone exposure and the lag (1day, 2d, 0-1, 1-2, 0-2). 3d average using kriging: FEV1 -0.12L (-0.19, -0.06), FVC -0.14 L (-0.22, -0.07)<br>Ages 15-64 had no significant relationships between ozone and lung function. 3d average using kriging: FEV1 0.05L (-0.01, 0.11) and FVC 0.02 (-0.05, 0.08)<br>In those over 65, no significant relationship was seen between ozone and FVC, but FEV1 showed significant decreases of 0.04-0.07 L at 0-day lag, 2-day lag, 0-1 and 0-2. 3d average using kriging: FEV1 -0.06L (-0.12, -0.01) and FVC -0.02 (-0.09, 0.05)	Mean ozone 40.9-42.2 ppb depending on the summary method. IQR 17.3ppb
Karakatsani et al 2017[28]	Cohort study in Greece	188 ten to 11-year-old elementary school students followed for 5 weeks in the 2013-2014 school year. Each child performed spirometry 5 times.	Personal exposure monitoring with Ogawa samplers	Increase of 10 $\mu g/m^3$ of weekly ozone associated with decreased FVC of -0.03L (-0.05, -0.01), FEV1 of -0.01 L (-0.03, 0.003) and 11.10% increase in FeNO (4.23, 18.43). Relationships with FEV1 and days with symptoms were not significant.	Personal ozone measurements means ranged from 4.7-10.8 $\mu g/m^3$
Kariisa et al 2015[29]	Cohort, re-analysis of a prior clinical trial	1218 COPD patients who had previously had lung volume reduction surgery (mean age 66y)	Measured daily and associated with participants at the zip code level. Lag0-3 were used for short term exposures and long term exposures were calculated for the interval since the last assessment (ranging 6-12 months)	Per 10 ppb of long term ozone, post bronchodilator FEV1 percent predicted decreased -0.227 (95% CI -0.539, 0.085) and FVC % predicted decreased -0.499 (95%CI -1.03,0.030).<br><br>Per 10 ppb of same-day ozone, post bronchodilator FEV1 percent predicted increased 4.94 (95% CI -7.92, 17.80) and FVC % predicted decreased -14.93 (95%CI -1.03,29.33).	Mean ozone 40 ppb

Online supplements are not copyedited prior to posting and the author(s) take full responsibility for the accuracy of all data.

Kurai et al 2018[30]	Longitudinal cohort in Japanese schoolchildren	276 school children (age 11-12y) followed with afternoon peak flow Sept-Oct 2016 and Jan-Feb 2017	Daily averages at the city level	A 13.6 ppb increase in same day ozone associated with a -3.45 L/min change in PEF (95% CI -4.64, -2.52), adjusted for $PM_{2.5}$<br><br>Ozone also associated with a small decrease at one day lag, but a small increase at the 2 day lag.	Mean ozone 33.8 ppb, IQR 13.6
Lepeule et al 2014[31]	Cohort study	776 men with lung function testing up to 4 times between 1999-2009	Measured at fixed monitoring sites from 4hour to 28 days prior to lung function testing.	Moving average of ozone from same day through 4 days prior associated with a 1.6% decrease in FEV1 (measured in L) per IQR of ozone. Other lags not significantly associated with ozone, and ozone not significantly associated with FVC (measured in L).<br><br>Estimated from figure 1. For a 5 day average (0-4 days) increase in one IQR of ozone FEV1 -1.5 % (-3.0, 0.25) and FVC -1.0% (-2.5 , 0.5).<br><br>Not included in forest plots because the results had to be estimated from the figures.	Mean ozone 47 $\mu g/m^3$, IQR 33 $\mu g/m^3$.
Li et al 2016[32]	Cohort study in New Taipei, Taiwan	59 children with asthma or allergies (mean age 11.3y, sd 2.5)	From a single Taiwan EPA monitoring station	From a two-pollutant model, adjusted for total particles, an IQR increase in ozone (19.8 ppb) lagged one day, was associated with a 0.09 L increase in FVC (-0.17, 0.36) and no change in FEV1 0.00 (-0.25, 0.25). Models adjusted for medication use.	Mean ozone was 32.7 ppb, IQR 19.8 ppb<br><br>Note that ozone results are in the supplement
Li et al 2018[33]	Cohort study of COPD patients in Beijing	43 patients (ages 58-81) that completed 215 home visits total	Hourly ozone obtained from nearby central monitoring stations (6 stations total, and participants were assigned the value from the closest station).	The largest effects were at the five day lag. For an IQR increase in ozone (adjusted for PM2.5), percent changes in participant's own mean FEV1 (in L) was -5.92% (95% CI -11.20, -0.64). No FVC results reported.<br><br>Note that they ran analyses with 24 h, 8h and 1h ozone averages and found that the 8h averages were associated with the strongest effect, but that both 1 and 24h averages showed a similar pattern.	Mean 8h max ozone was 80.3 $\mu g/m^3$, with an IQR of 86.9
Liu et al 2018[34]	Cohort of adults in Taipei-Keelung metropolitan area, Taiwan	100 healthy non-smoking adults 20-64 years old	From stations of the local EPA monitoring network, matched to the closest station to the participant's residence (within 10 km)	For an IQR increment of ozone lagged one day, FEV1 decreased -2.0% (-3.3, -0.7) in a single pollutant model.	Mean ozone 13.1 ppb, with maximum of 21.3 ppb. IQR of 7.4 ppb

Online supplements are not copyedited prior to posting and the author(s) take full responsibility for the accuracy of all data.

Martenies et al 2020[35]	Cohort of Dairy workers in Colorado (at 4 different dairy operations)	36 workers at high risk of bioaerosol exposure did pre/post shift spirometry 1-8 times each	From an EPA monitor within 50 km of the dairy	With a doubling in ozone exposure, FVC % predicted pre/post increased by 0.44 (-2.27, 3.15) and FEV1 increased by 0.80 (-2.43, 4.04), and PEF increased by 4.86 (-6.80, 16.53).	Geometric mean ozone 50 ppb (ranged 30-80 ppb)
Moreno-Macias et al 2013[36]	Cohort using children from 2 prior studies (a cohort and a clinical trial) in Mexico City	257 children with asthma genotyped for GSTM1	From the monitoring station closest to the child's home	Overall, acute ozone not significantly related to FEF_{25-75}. GSTM1 genotype or copy numbers not related to lung function response to ozone. Vitamin C intake status also not related.<br><br>In the persistent asthmatics subgroup, those with no GSTM1 copies did have a significant ozone related decrement in FEF_{25-75}, -36.6 ml/s (p=0.05) per 60 ppb ozone.	IQR of ozone 60 ppb. Mean ozone 96.9 ppb
Neophytou et al 2016[37]	Cohort study in 5 regions of the US	1449 Latino and 519 African American children with asthma	Calculated from the inverse distance squared weighting of the four monitors closest to the participants' residence.	Lifetime, first year of life, monthly or acute ozone exposure not significantly related to lung function.<br><br>5 ppb increase in lifetime ozone associated with -0.65% FEV1 measured in L (-1.96, 0.68)	Average ozone ranged from 15-40 ppb depending on metro region.
Pasalic et al 2016[38]	Cross-sectional study of high school students involved in extracurricular activities in Atlanta, GA	Convenience sample of 126 students from 2 high schools	Ozone measured on site during sports practice/activity session, calculated inhaled doses using minute ventilation from measured breathing rates	Contradictory results reported in the tables, so this study has been left off our forest plots.<br><br>For example in the single pollutant model, for the variable associating ozone with change in FEV1 before/after activity, the sign is different between the raw coefficient and the coefficient scaled per IQR of pollutant.<br><br>In the multipollutant model, the raw coefficient given for ozone does not match the coefficient scaled to an IQR when using the IQR reported in the text.	Median inhaled dose of ozone was 249.8 μg, with an IQR of 345.64 μg.

Online supplements are not copyedited prior to posting and the author(s) take full responsibility for the accuracy of all data.

Paulin et al 2019[39]	Large cross sectional study in multiple sites around the US	1874 adults 40-80 years old who were current or former smokers	10 year ozone concentration estimated with spatiotemporal modeling to participant home addresses	5 ppb increase in ozone concentration (adjusted for pm2.5) was associated with lower percent predicted FEV1 -2.86% (95% CI -5.58,-0.15).<br><br>No FVC results reported.<br><br>Their sensitivity model (using polynomials) suggest non-linearity in the relationship with a steeper decline in FEV1 at lower ozone concentrations.	All ozone estimates less than 50 ppb.
Rice et al 2013[40]	Participants in a long-term multi-generational study in Framingham, MA	3262 non-smoking participants in the offspring and third generation Framingham cohorts who had 1-2 spirometry measurements in the specified intervals	Ozone extracted from state monitoring network and only available April to September, and measurements from all Boston sites were averaged. This study intentionally excluded observations that exceeded the AQI moderate level for the pollutant (less than or equal to 75 ppb for ozone at the time)	For a 10 ppb increase in ozone with a 1 day lag, participants had -17.4 mL lower FEV1 (95% CI -30.9, -4.0).<br><br>FVC effect estimated from the figure and thus not included in our forest plots, -11 mL (95% -26, 5).<br><br>There were significantly stronger ozone effects seen in obese participants and in men compared to women.	All ozone measurements were (by definition for this study) less than 75 ppb. Mean ozone was 28.7 ppb
Samoli et al 2017[41]	Cohort followed for 5 weeks each during the school year	186 10-11 year olds in Athens and Thessalonaki, Greece, attending schools in either high or low ozone areas (within 2 km of a monitoring station)	Data used fixed monitoring at schools as well as personal measurements.	Per 10 μg/m^3 of same-day ozone measured at the fixed school sites and adjusted for PM_{10}, PEF not substantially different, -0.01 (-0.6, 0.6).<br><br>Using personal same-day ozone monitoring, again adjusted for PM_{10}, effect was 0.91 (-1.50, 3.32).	Note that this is the same cohort of children described in the Dimakopolou and Karakatsani papers, with a different ozone averaging time.
Tsui et al 2018[42]	Nationwide, cross-sectional school-based survey in Taiwan	1016 school children, mean age 11.9 +/- 2.4 years	Lifetime modeled O3 at residential addresses using EPA monitoring data	FEV1 % predicted -0.93 (-1.53, -0.34) per 1 ppb O3, becomes not statistically significant once PM2.5 included in model (though still significant if two pollutant models are used with other pollutants). Controlling for NO2, FEV1 % predicted -0.91 (-1.68, -0.14) and FVC% predicted -0.84 (-1.62, -0.06)	Mean ozone approximately 26 ppb

Online supplements are not copyedited prior to posting and the author(s) take full responsibility for the accuracy of all data.

Urman et al 2014[43]	Cross- sectional analysis of a cohort study in Los Angeles	1811 children completed lung function testing	Regional ozone from community monitoring stations averaged over the six years prior to lung function testing	22.7 ppb increase ozone associated with -3.1% decrease in FEV1 measured in mL (-5.24, -0.91), but no relationship with FVC in mL -0.31% (-3.11, 2.57).	Mean ozone 22.7 ppb
Usemann et al 2019[44]	Prospective birth cohort in Switzerland	304 healthy term infants followed to age 6 years	Combination of annual dispersion models and spatial temporal models from local monitor data, matched to residential addresses.	For a 14.5 µg/m³ increase in ozone exposure during pregnancy, FEV1 decreased -20.4 mL (-48.5, 7.7).<br><br>For a 8.7 µg/m³ increase in ozone exposure during the first year of life, FEV1 decreased -16.5 mL (-45.0, 11.9).<br><br>For a 6.0 µg/m³ increase in ozone exposure during the sixth year of life, FEV1 increased 4.6 mL (-20.7, 29.9).<br><br>For a 6.0 µg/m³ increase in ozone exposure from birth until age six, FEV1 decreased -21.2 mL (-49.1, 6.8).<br><br>For a 50 µg/m³ increase in ozone exposure during the prior two weeks, FEV1 decreased -40.7 (-72.9, -8.5).<br><br>FVCs not reported.	Mean ozone levels were 84-88 µg/m³.
Wang et al 2019[45]	Multi-Ethnic Study of Atherosclerosis (MESA) Air and Lung Studies in the US	Of 7071 study participants who were 45-84 years at recruitment, 3636 had at least one spirometry assessment, and 2772 of those had a second.	Annual air pollution assigned to residential address using spatio-temporal models.	Per 3 ppb of ozone for the year prior to the baseline assessment, FEV1 decreased -15.31 mL (95% -30.13, -0.5) and FVC -27.19 mL (95% CI -48.18, -6.20).<br>Multi-pollutant results for the baseline estimate not reported.<br><br>Using ozone averaged over the follow up interval (median 10 years) and controlling for both PM2.5 and NOx, FEV1 decreased -15.95 mL (95% CI -31.29, -0.60 and FVC decreased -31.44 mL (95% CI -57.86, -5.02)	Mean ozone over follow up ranged from 15-25.7 ppb among the different cities. Mean ozone at baseline ranged 14-25.2.

Online supplements are not copyedited prior to posting and the author(s) take full responsibility for the accuracy of all data.

Xing et al 2020[46]	Seven Northeastern Cities Study, a large cross-sectional study in China	6740 children, aged 7-14	Estimated from municipal monitoring stations. Four year mean ozone used as a proxy for long term exposure	In normal weight children, IQR of ozone associated with a decrease of -12.84 mL FVC (-17.19, -8.50); -8.33 mL FEV1 (-12.12, -4.54) and -27.47 mL/s PEF (-36.24, -18.71).<br><br>In overweight children, IQR of ozone associated with a decrease of -20.30 mL FVC (-30.51, -10.09); -14.04 mL FEV1 (-23.09, -5.00) and -27.26 mL/s PEF (-47.97, -6.55).<br><br>In obese children, IQR of ozone associated with a decrease of -21.80 mL FVC (-34.06, -9.53); -17.83 mL FEV1 (-28.11, -7.54) and -52.14 mL/s PEF (-74.35, -29.92).<br><br>This study is not in the figures because it reports the same data as Zhang 2019 and Zeng 2016.	IQR of ozone was 46.3 μg/m³. Mean ozone was 96.8 μg/m³.
Yoda et al 2014[47]	Cohort study	21 healthy young women studied for two weeks in the summer in Tokyo. Measurements taken 4 times over that period.	Air pollutant data from the Atmospheric environmental Regional Observation System of the Ministry of the Environment, Japan. Ozone summarized as 24-hour averages.	10 ppb increase in Ozone significantly associated with decreased exhaled breath condensate pH, with a decrease of 0.02-0.07 depending on the lag used (same day or average including up to five days before). Ozone not significantly related to FeNO, peak expiratory flow or FEV1. 10 ppb change in 4 day average ozone adjusted for NO2 associated with 0.00 L increase in FEV1 (-0.15, 0.15)	Ozone ranged roughly 10-60 ppb
Yoda et al 2017[48]	Longitudinal Cohort	43 healthy students (15-16 years) on Seto Island, Japan followed for the month of May 2014, with daily lung function assessments	Ogawa passive sampler for ozone collected both indoors and outdoors every 24 hours at the school site, 24 hours prior to lung function was used in the models	IQR increase in indoor ozone adjusted for PM2.5 is associated with 7.77 L/min decrease in peak expiratory flow (-12.81, -2.73) and -19.35 mL FEV1 (-54.83, 16.14). These effects largely driven by the decrease in children with asthma.	IQR of indoor ozone was 11 ppb. Outdoor ozone mean 44.6 ppb, indoor 15.9 ppb.
Zeng et al 2016[49]	Seven Northeastern Cities Study, a large cross-sectional study in China	6740 children, aged 7-14	Estimated from municipal monitoring stations. Four year mean ozone used as a proxy for long term exposure	Per IQR increase in four year average ozone, FVC decreased -21.87 (SE 9.30), FEV1 decreased -21.13 ml (SE 8.49), and -29.69 mL/s PEF (SE 17.89).<br>Effects were larger in males than females.	IQR of 46.3 μg/m³.
C. Zhang et al 2019 (JAMA Netw Open)[50]	Seven Northeastern Cities Study, a large cross-sectional study in China	6740 children, aged 7-14	Estimated from municipal monitoring stations. Four year mean ozone used as a proxy for long term exposure	For one IQR increase in ozone, FVC decreased -23.09 mL (-42.88, -3.30) among children who were not breastfed as infants compared to -8.00 mL (-23.24, 7.23) among those that were breastfed.	IQR of ozone was 46.3 μg/m³. Mean ozone was 106.9 μg/m³.

Online supplements are not copyedited prior to posting and the author(s) take full responsibility for the accuracy of all data.

				For one IQR increase in ozone, FEV1 decreased -17.92 mL (-34.84, -0.99) among children who were not breastfed as infants compared to -8.60 mL (-22.48, 5.29) among those that were breastfed.<br><br>For one IQR increase in ozone, PEF decreased -32.07 mL/s (-67.69 -3.55) among children who were not breastfed as infants compared to -25.23 mL (-50.90, 0.43) among those that were breastfed.<br>This study is not in the figures because it reports the same data as Xing 2020 and Zeng 2016.	
J. Zhang et al 2019 (Env Sci Res Poll Int)[51]	Cross-sectional study in China	40 non-smoking elderly volunteers (over age 50)	24h of personal ozone measurements	For a 10 μg/m^3 increase in ozone FVC decreased by -0.14 L (95% CI -0.02, -0.26) FEV1 by -0.11 L (95% CI -0.01, -0.21), and PEF increased by 0.01 L (0.02, -0.26).<br><br>(all corrected for PM2.5)	Mean ozone 28.40 , range 6.8-77.6 μg/m^3
Zhang et al 2020 (Environ Geochem Health)[52]	Cohort study in Tianjin, China	198 healthy primary school students in grades 3-5 (age 8-13) from 9 schools, assessed 5 times each	Land use regression model to predict level as participants' homes.	10 μg/m^3 increase in ozone over 2 days (lags0-1) associated with decrease of 21.09 % in FVC measured in L (-25.54, -16.58), FEV1 in L of -20.87 % (-24.88, -16.80) and PEF in L -24.67 % (-31.22, -18.06). Results for the 3 day averages were similar.	Mean ozone 27.73 , with IQR of 13.50 μg/m^3
Zhou et al 2016[53]	Wuhan-Zhuhai Cohort in China	1694 non-smoking women (mean age 56, range 21-84)	Calculated up to 8 day moving averages, using national air quality monitoring and calculating at the community level (4 communities total.)	10 μg/m^3 increase in ozone using same day average associated with a -5 mL change in FVC (95% CI -11,1) and a -3 mL change in FEV1 (95% CI -8, 2),<br>All point estimates and CI estimated from the figures as these are not reported in the text. Thus this paper is not included in our forest plots.	Mean ozone 50.73 μg/m^3 and IQR 52.7

Online supplements are not copyedited prior to posting and the author(s) take full responsibility for the accuracy of all data.

References

1. Adman MA, Hashim JH, Manaf MRA, Norback D. Associations between air pollutants and peak expiratory flow and fractional exhaled nitric oxide in students. *The International Journal of Tuberculosis and Lung Disease* 2020;24(2):189–195.

2. Altuğ H, Gaga EO, Döğeroğlu T, et al. Effects of air pollution on lung function and symptoms of asthma, rhinitis and eczema in primary school children. *Environ Sci Pollut Res* 2013;20(9):6455–6467.

3. Amadeo B, Robert C, Rondeau V, et al. Impact of close-proximity air pollution on lung function in schoolchildren in the French West Indies. *BMC Public Health* 2015;15(1):45.

4. Barbone F, Catelan D, Pistelli R, et al. A Panel Study on Lung Function and Bronchial Inflammation among Children Exposed to Ambient SO2 from an Oil Refinery. *International Journal of Environmental Research and Public Health* 2019;16(6):1057.

5. Barone-Adesi F, Dent JE, Dajnak D, et al. Long-Term Exposure to Primary Traffic Pollutants and Lung Function in Children: Cross-Sectional Study and Meta-Analysis. *PLoS ONE* 2015;10(11):e0142565.

6. Benka-Coker W, Hoskovec L, Severson R, Balmes J, Wilson A, Magzamen S. The joint effect of ambient air pollution and agricultural pesticide exposures on lung function among children with asthma. *Environmental Research* 2020;190:109903.

7. Benmerad M, Slama R, Botturi K, et al. Chronic effects of air pollution on lung function after lung transplantation in the Systems prediction of Chronic Lung Allograft Dysfunction (SysCLAD) study. *Eur Respir J* 2017;49(1):1600206.

8. Bräuner EV, Karottki DG, Frederiksen M, et al. Residential ozone and lung function in the elderly. *Indoor and Built Environment* 2016;25(1):93–105.

9. Chang L-T, Hong G-B, Weng S-P, et al. Indoor ozone levels, houseplants and peak expiratory flow rates among healthy adults in Taipei, Taiwan. *Environment International* 2019;122:231–236.

10. Chen R, Zhao A, Chen H, et al. Cardiopulmonary Benefits of Reducing Indoor Particles of Outdoor Origin: A Randomized, Double-Blind Crossover Trial of Air Purifiers. *Journal of the American College of Cardiology* 2015;65(21):2279–2287.

11. Chen B-Y, Chen C-H, Chuang Y-C, et al. Schoolchildren's antioxidation genotypes are susceptible factors for reduced lung function and airway inflammation caused by air pollution. *Environmental Research* 2016;149:145–150.

12. Dales RE, Cakmak S. Does Mental Health Status Influence Susceptibility to the Physiologic Effects of Air Pollution? A Population Based Study of Canadian Children. *PLoS ONE* 2016;11(12):e0168931.

13. Dauchet L, Hulo S, Cherot-Kornobis N, et al. Short-term exposure to air pollution: Associations with lung function and inflammatory markers in non-smoking, healthy adults. *Environment International* 2018;121:610–619.

14. Day DB, Xiang J, Mo J, et al. Association of Ozone Exposure With Cardiorespiratory Pathophysiologic Mechanisms in Healthy Adults. *JAMA Intern Med* 2017;177(9):1344.

15. Dimakopoulou K, Douros J, Samoli E, et al. Long-term exposure to ozone and children's respiratory health: Results from the RESPOZE study. *Environmental Research* 2020;182:109002.

16. Fernández-Plata R, Rojas-Martínez R, Martínez-Briseño D, García-Sancho C, Pérez-Padilla R. Effect of Passive Smoking on the Growth of Pulmonary Function and Respiratory Symptoms in Schoolchildren. :9.

17. Fuertes E, Bracher J, Flexeder C, et al. Long-term air pollution exposure and lung function in 15 year-old adolescents living in an urban and rural area in Germany: The GINIplus and LISAplus cohorts. *International Journal of Hygiene and Environmental Health* 2015;218(7):656–665.

18. Gauderman WJ, Urman R, Avol E, et al. Association of Improved Air Quality with Lung Development in Children. *New England Journal of Medicine* 2015;372(10):905–913.

19. Gilliland F, Avol E, McConnell R, et al. Effects of Policy-Driven Air Quality Improvements on Children's Respiratory Health. :98.

20. Hasunuma H, Yamazaki S, Tamura K, et al. Association between daily ambient air pollution and respiratory symptoms in children with asthma and healthy children in western Japan. *Journal of Asthma* 2018;55(7):712–719.

21. Hernandez ML, Dhingra R, Burbank AJ, et al. Low-level ozone has both respiratory and systemic effects in African American adolescents with asthma despite asthma controller therapy. *Journal of Allergy and Clinical Immunology* 2018;142(6):1974-1977.e3.

22. Huang J, Song Y, Chu M, et al. Cardiorespiratory responses to low-level ozone exposure: The inDoor Ozone Study in childrEn (DOSE). *Environment International* 2019;131:105021.

23. Hwang B-F, Chen Y-H, Lin Y-T, Wu X-T, Leo Lee Y. Relationship between exposure to fine particulates and ozone and reduced lung function in children. *Environmental Research* 2015;137:382–390.

24. Ierodiakonou D, Zanobetti A, Coull BA, et al. Ambient air pollution, lung function, and airway responsiveness in asthmatic children. *Journal of Allergy and Clinical Immunology* 2016;137(2):390–399.

25. Int Panis L, Provost EB, Cox B, et al. Short-term air pollution exposure decreases lung function: a repeated measures study in healthy adults. *Environ Health* 2017;16(1):60.

26. Johannson KA, Vittinghoff E, Morisset J, et al. Air Pollution Exposure Is Associated With Lower Lung Function, but Not Changes in Lung Function, in Patients With Idiopathic Pulmonary Fibrosis. *Chest* 2018;154(1):119–125.

27. Jung S-W, Lee K, Cho Y-S, et al. Association by Spatial Interpolation between Ozone Levels and Lung Function of Residents at an Industrial Complex in South Korea. *IJERPH* 2016;13(7):728.

28. Karakatsani A, Samoli E, Rodopoulou S, et al. Weekly Personal Ozone Exposure and Respiratory Health in a Panel of Greek Schoolchildren. *Environ Health Perspect* 2017;125(7):077017.

29. Kariisa M, Foraker R, Pennell M, et al. Short- and Long-Term Effects of Ambient Ozone and Fine Particulate Matter on the Respiratory Health of Chronic Obstructive Pulmonary Disease Subjects. *Archives of Environmental & Occupational Health* 2015;70(1):56–62.

30. Kurai J, Noma H, Sano H, Iwata K, Tohda Y, Watanabe M. Association of short-term ozone exposure with pulmonary function and respiratory symptoms in schoolchildren:A panel study in a western Japanese city. *J Med Invest* 2018;65(3.4):236–241.

31. Lepeule J, Bind M-AC, Baccarelli AA, et al. Epigenetic Influences on Associations between Air Pollutants and Lung Function in Elderly Men: The Normative Aging Study. *Environmental Health Perspectives* 2014;122(6):566–572.

Online supplements are not copyedited prior to posting and the author(s) take full responsibility for the accuracy of all data.

32. Li Y-R, Feng L-T, Chen B-Y, et al. Association of urban particle numbers and sources with lung function among children with asthma or allergies. *Science of The Total Environment* 2016;542:841–844.

33. Li H, Wu S, Pan L, et al. Short-term effects of various ozone metrics on cardiopulmonary function in chronic obstructive pulmonary disease patients: Results from a panel study in Beijing, China. *Environmental Pollution* 2018;232:358–366.

34. Liu J-Y, Hsiao T-C, Lee K-Y, Chuang H-C, Cheng T-J, Chuang K-J. Association of ultrafine particles with cardiopulmonary health among adult subjects in the urban areas of northern Taiwan. *Science of The Total Environment* 2018;627:211–215.

35. Martenies SE, Schaeffer JW, Erlandson G, et al. Associations Between Bioaerosol Exposures and Lung Function Changes Among Dairy Workers in Colorado. *Journal of Occupational and Environmental Medicine* 2020;62(6):424–430.

36. Moreno-Macías H, Dockery DW, Schwartz J, et al. Ozone exposure, vitamin C intake, and genetic susceptibility of asthmatic children in Mexico City: a cohort study. *Respir Res* 2013;14(1):14.

37. Neophytou AM, White MJ, Oh SS, et al. Air Pollution and Lung Function in Minority Youth with Asthma in the GALA II (Genes–Environments and Admixture in Latino Americans) and SAGE II (Study of African Americans, Asthma, Genes, and Environments) Studies. *Am J Respir Crit Care Med* 2016;193(11):1271–1280.

38. Pasalic E, Hayat M, Greenwald R. Air pollution, physical activity, and markers of acute airway oxidative stress and inflammation in adolescents. *JGPHA* [Internet] 2016 [cited 2021 May 8];6(5). Available from: https://digitalcommons.georgiasouthern.edu/jgpha/vol6/iss5/19

39. Paulin LM, Gassett AJ, Alexis NE, et al. Association of Long-term Ambient Ozone Exposure With Respiratory Morbidity in Smokers. *JAMA Intern Med* 2020;180(1):106.

40. Rice MB, Ljungman PL, Wilker EH, et al. Short-Term Exposure to Air Pollution and Lung Function in the Framingham Heart Study. *Am J Respir Crit Care Med* 2013;188(11):1351–1357.

41. Samoli E, Dimakopoulou K, Evangelopoulos D, et al. Is daily exposure to ozone associated with respiratory morbidity and lung function in a representative sample of schoolchildren? Results from a panel study in Greece. *J Expo Sci Environ Epidemiol* 2017;27(3):346–351.

42. Tsui H-C, Chen C-H, Wu Y-H, Chiang H-C, Chen B-Y, Guo YL. Lifetime exposure to particulate air pollutants is negatively associated with lung function in non-asthmatic children. *Environmental Pollution* 2018;236:953–961.

43. Urman R, McConnell R, Islam T, et al. Associations of children's lung function with ambient air pollution: joint effects of regional and near-roadway pollutants. *Thorax* 2014;69(6):540–547.

44. Usemann J, Decrue F, Korten I, et al. Exposure to moderate air pollution and associations with lung function at school-age: A birth cohort study. *Environment International* 2019;126:682–689.

45. Wang M, Aaron CP, Madrigano J, et al. Association Between Long-term Exposure to Ambient Air Pollution and Change in Quantitatively Assessed Emphysema and Lung Function. *JAMA* 2019;322(6):546.

46. Xing X, Hu L, Guo Y, et al. Interactions between ambient air pollution and obesity on lung function in children: The Seven Northeastern Chinese Cities (SNEC) Study. *Science of The Total Environment* 2020;699:134397.

47. Yoda Y, Otani N, Sakurai S, Shima M. Acute Effects of Summer Air Pollution on Pulmonary Function and Airway Inflammation in Healthy Young Women. *Journal of Epidemiology* 2014;24(4):312–320.

48. Yoda Y, Takagi H, Wakamatsu J, et al. Acute effects of air pollutants on pulmonary function among students: a panel study in an isolated island. *Environ Health Prev Med* 2017;22(1):33.

49. Zeng X-W, Vivian E, Mohammed KA, et al. Long-term ambient air pollution and lung function impairment in Chinese children from a high air pollution range area: The Seven Northeastern Cities (SNEC) study. *Atmospheric Environment* 2016;138:144–151.

50. Zhang C, Guo Y, Xiao X, et al. Association of Breastfeeding and Air Pollution Exposure With Lung Function in Chinese Children. *JAMA Netw Open* 2019;2(5):e194186.

51. Zhang J, Sun H, Chen Q, Gu J, Ding Z, Xu Y. Effects of individual ozone exposure on lung function in the elderly: a cross-sectional study in China. *Environ Sci Pollut Res* 2019;26(12):11690–11695.

52. Zhang J, Feng L, Hou C, Gu Q. How the constituents of fine particulate matter and ozone affect the lung function of children in Tianjin, China. *Environ Geochem Health* 2020;42(10):3303–3316.

53. Zhou Y, Liu Y, Song Y, et al. Short-term Effects of Outdoor Air Pollution on Lung Function among Female Non-smokers in China. *Sci Rep* 2016;6(1):34947.

Online supplements are not copyedited prior to posting and the author(s) take full responsibility for the accuracy of all data.

e-Appendix 2
NEWCASTLE - OTTAWA QUALITY ASSESSMENT SCALE
CASE CONTROL STUDIES

Note: A study can be awarded a maximum of one star for each numbered item within the Selection and Exposure categories. A maximum of two stars can be given for Comparability.

Notes for our adaptations of the scale are in italics.

Selection

1) Is the case definition adequate?
   a) yes, with independent validation ✸
   b) yes, eg record linkage or based on self reports
   c) no description

2) Representativeness of the cases
   a) consecutive or obviously representative series of cases ✸
   b) potential for selection biases or not stated

3) Selection of Controls
   a) community controls ✸
   b) hospital controls
   c) no description

For this item, all case-crossover studies were marked as having 'community controls' as these controls ARE representative of the study base from which cases are drawn, which is the intent of this question.

4) Definition of Controls
   a) no history of disease (endpoint) ✸
   b) no description of source

For this item, the control days from a case-crossover study were marked as having 'no history of disease' only if (A) the outcome was one which could not occur on multiple days (eg. death), or (B) the authors documented that there was verification of no disease on the control days in some way. Otherwise, the control days from other case-crossover studies would be marked as 'no description of source'.

Comparability

1) Comparability of cases and controls on the basis of the design or analysis
   a) study controls for *SES* ✸

For this item, all case-crossover studies were marked as controlling for SES because they control for within subject factors by design. All other case-control studies needed to include SES in the analysis in which ozone was studied in order to receive credit for this item.

   b) study controls for *other air pollutants*✸

Online supplements are not copyedited prior to posting and the author(s) take full responsibility for the accuracy of all data.

For this item, a study only got credit for controlling for other air pollutants if they were included in two or multi-pollutant models with ozone, NOT if the study assessed multiple pollutants individually.

Exposure

1) Ascertainment of exposure

a) secure record (eg surgical records) ✸

b) structured interview where blind to case/control status ✸

c) interview not blinded to case/control status

d) written self report or medical record only

e) no description

Much of the variability in exposures in this body of literature relates to whether exposures were assessed at the individual level (either with individual or household monitoring or with residential modelling using geographic techniques) or if one exposure measurement was used for many participants in the study (ie. a central city monitor used for many or all participants). To represent this variability in this question, any individual ozone measurement or modelled value was coded as option a. Ozone measurements at the city level or higher were coded as d.

2) Same method of ascertainment for cases and controls

a) yes ✸

b) no

3) Non-Response rate

a) same rate for both groups ✸

b) non respondents described

c) rate different and no designation

For this item, any studies that did not mention non-response rates were marked as 'rate different and no designation'. A case-crossover study was marked as having 'same rate for both groups' or 'non respondents described' only if the authors explicitly stated how many case and control days had to be excluded due to inability to match exposure data. Otherwise, other case-crossover studies would be marked as 'rate different and no designation'.

Online supplements are not copyedited prior to posting and the author(s) take full responsibility for the accuracy of all data.

NEWCASTLE - OTTAWA QUALITY ASSESSMENT SCALE
COHORT STUDIES

Note: A study can be awarded a maximum of one star for each numbered item within the Selection and Outcome categories. A maximum of two stars can be given for Comparability

Notes for our adaptations of the scale are in italics.

Selection

1) Representativeness of the exposed cohort
   a) truly representative of the average _______________ in the community ✸
   b) somewhat representative of the average ______________ in the community ✸
   c) selected group of users eg nurses, volunteers
   d) no description of the derivation of the cohort

Note that due to the wide inclusion criteria for this review, the population that was intended to be represented for a given study varied, but was generally a community-based population of people in the studied age group.

2) Selection of the non exposed cohort
   a) drawn from the same community as the exposed cohort ✸
   b) drawn from a different source
   c) no description of the derivation of the non exposed cohort

3) Ascertainment of exposure
   a) secure record (eg surgical records) ✸
   b) structured interview ✸
   c) written self report
   d) no description

As for case-control studies, much of the variability in exposures in this body of literature relates to whether exposures were assessed at the individual level (either with individual or household monitoring or with residential modelling using geographic techniques) or if one exposure measurement was used for many participants in the study (ie. a central city monitor used for many or all participants). To represent this variability in this question, any individual ozone measurement or modelled value was coded as option a. Ozone measurements at the city level or higher were coded as c.

4) Demonstration that outcome of interest was not present at start of study
   a) yes ✸
   b) no

Online supplements are not copyedited prior to posting and the author(s) take full responsibility for the accuracy of all data.

Comparability

1) Comparability of cases and controls on the basis of the design or analysis
   a) study controls for *SES* ✵

Cohort studies needed to include SES in the analysis in which ozone was studied in order to receive credit for this item.

   b) study controls for *other air pollutants*✵

For this item, a study only got credit for controlling for other air pollutants if they were included in two or multi-pollutant models with ozone, NOT if the study assessed multiple pollutants individually.

Outcome

1) Assessment of outcome
   a) independent blind assessment ✵
   b) record linkage ✵
   c) self report
   d) no description

2) Was follow-up long enough for outcomes to occur
   a) yes (select an adequate follow up period for outcome of interest) ✵
   b) no

3) Adequacy of follow up of cohorts
   a) complete follow up - all subjects accounted for ✵
   b) subjects lost to follow up unlikely to introduce bias - small number lost - > *85* % follow up, or description provided of those lost) ✵
   c) follow up rate < *85* % and no description of those lost
   d) no statement

ADAPTED NEWCASTLE - OTTAWA QUALITY ASSESSMENT SCALE
CROSS-SECTIONAL STUDIES

Note: A study can be awarded a maximum of one star for each numbered item within the Selection and Outcome categories. A maximum of two stars can be given for Comparability

Note- This is adapted from the Cohort tool, by excluding only those questions related to cohort follow up.

Selection

1) Representativeness of the exposed cohort
   - a) truly representative of the average _______________ in the community ✵
   - b) somewhat representative of the average ______________ in the community ✵
   - c) selected group of users eg nurses, volunteers
   - d) no description of the derivation of the cohort

Note that due to the wide inclusion criteria for this review, the population that was intended to be represented for a given study varied, but was generally a community-based population of people in the studied age group.

2) Selection of the non exposed cohort
   - a) drawn from the same community as the exposed cohort ✵
   - b) drawn from a different source
   - c) no description of the derivation of the non exposed cohort

3) Ascertainment of exposure
   - a) secure record (eg surgical records) ✵
   - b) structured interview ✵
   - c) written self report
   - d) no description

As for case-control studies, much of the variability in exposures in this body of literature relates to whether exposures were assessed at the individual level (either with individual or household monitoring or with residential modelling using geographic techniques) or if one exposure measurement was used for many participants in the study (ie. a central city monitor used for many or all participants). To represent this variability in this question, any individual ozone measurement or modelled value was coded as option a. Ozone measurements at the city level or higher were coded as c.

4) Demonstration that outcome of interest was not present at start of study
   - a) yes ✵
   - b) no

Online supplements are not copyedited prior to posting and the author(s) take full responsibility for the accuracy of all data.

Comparability

1) Comparability of cases and controls on the basis of the design or analysis
   a) study controls for *SES* ✸

Cohort studies needed to include SES in the analysis in which ozone was studied in order to receive credit for this item.

   b) study controls for *other air pollutants*✸

For this item, a study only got credit for controlling for other air pollutants if they were included in two or multi-pollutant models with ozone, NOT if the study assessed multiple pollutants individually.

Outcome

1) Assessment of outcome
   a) independent blind assessment ✸
   b) record linkage ✸
   c) self report
   d) no description

Online supplements are not copyedited prior to posting and the author(s) take full responsibility for the accuracy of all data.

ADAPTED NEWCASTLE - OTTAWA QUALITY ASSESSMENT SCALE ECOLOGIC STUDIES

Note: A study can be awarded a maximum of one star for each numbered item within the Selection and Outcome categories. A maximum of two stars can be given for Comparability

Note- This is adapted from the Cohort tool, by including only those questions related to comparability, as ecologic designs are known to have high risk of bias in the domains of selection and outcome.

Comparability

1) Comparability of cases and controls on the basis of the design or analysis
   a) study controls for *SES* ✵

Cohort studies needed to include SES in the analysis in which ozone was studied in order to receive credit for this item.

   b) study controls for *other air pollutants*✵

For this item, a study only got credit for controlling for other air pollutants if they were included in two or multi-pollutant models with ozone, NOT if the study assessed multiple pollutants individually.

ROBINS-I

Assessments using the ROBINS-I tool followed the detailed guidance available from:
https://www.riskofbias.info/welcome/home/current-version-of-robins-i/robins-i-detailed-guidance-2016

(See separate file for e-Appendix 3.)

Online supplements are not copyedited prior to posting and the author(s) take full responsibility for the accuracy of all data.

e-Appendix 4

Figure 1. Risk of bias visualization for all the studies, using the results from the Newcastle-Ottawa Scale. Each subscale was rescaled to be out of 4 points and rounded to the nearest integer. Subscale scores of 0-1 were assigned "critical", 2 were assigned "high risk", 3 were assigned "unclear risk", 4 were assigned "low risk". For the overall scores, scores of 0-2 were assigned "critical", 3-4 were assigned "high risk", 5-6 were assigned "unclear risk", 7-8 were assigned "low risk".

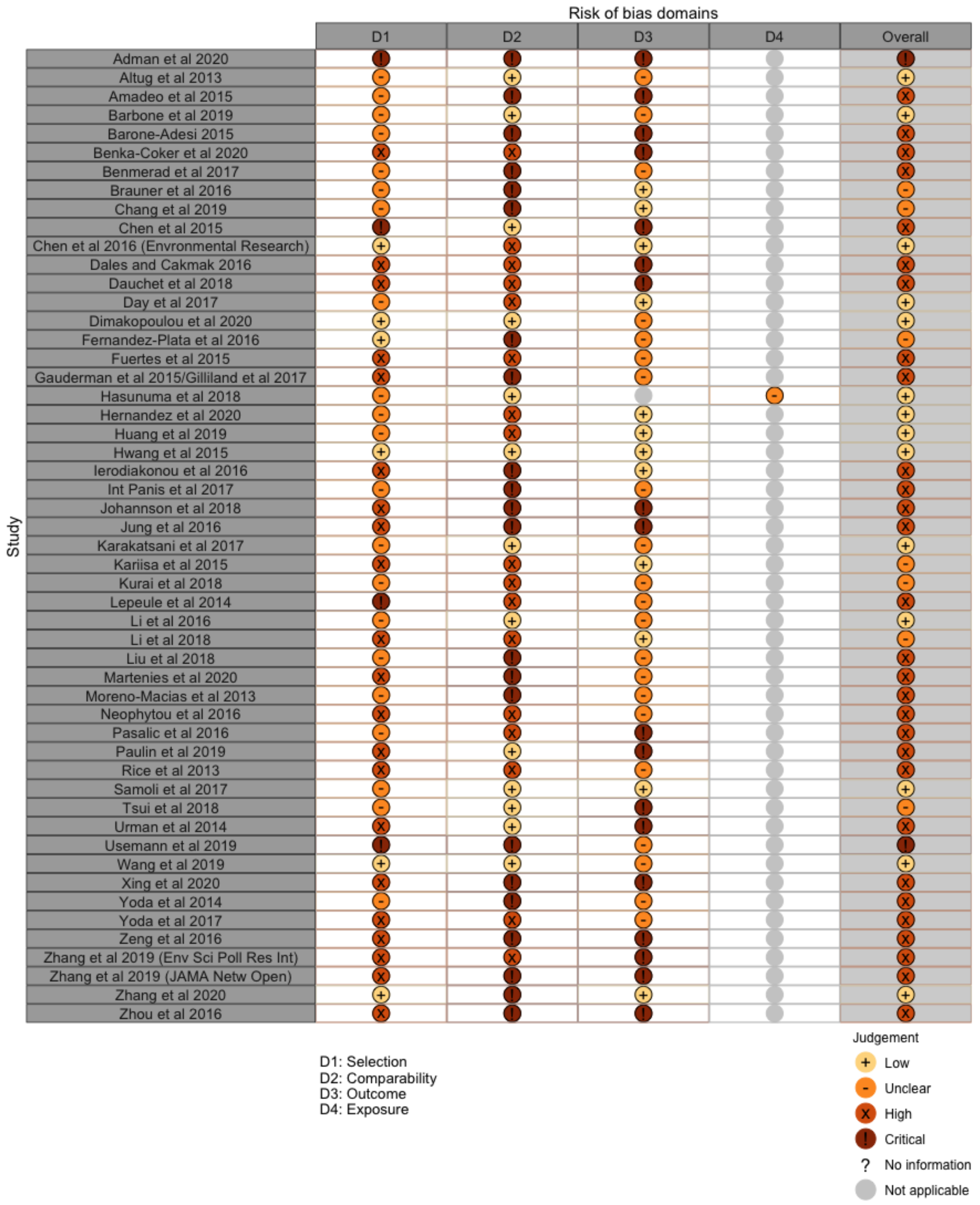

Online supplements are not copyedited prior to posting and the author(s) take full responsibility for the accuracy of all data.

Figure 2. Risk of bias visualization for all the studies, using the results from the Risk of Bias in Non-randomized Studies of Interventions (ROBINS-I) tool, which grades each of these domains using the scale presented in the figure.

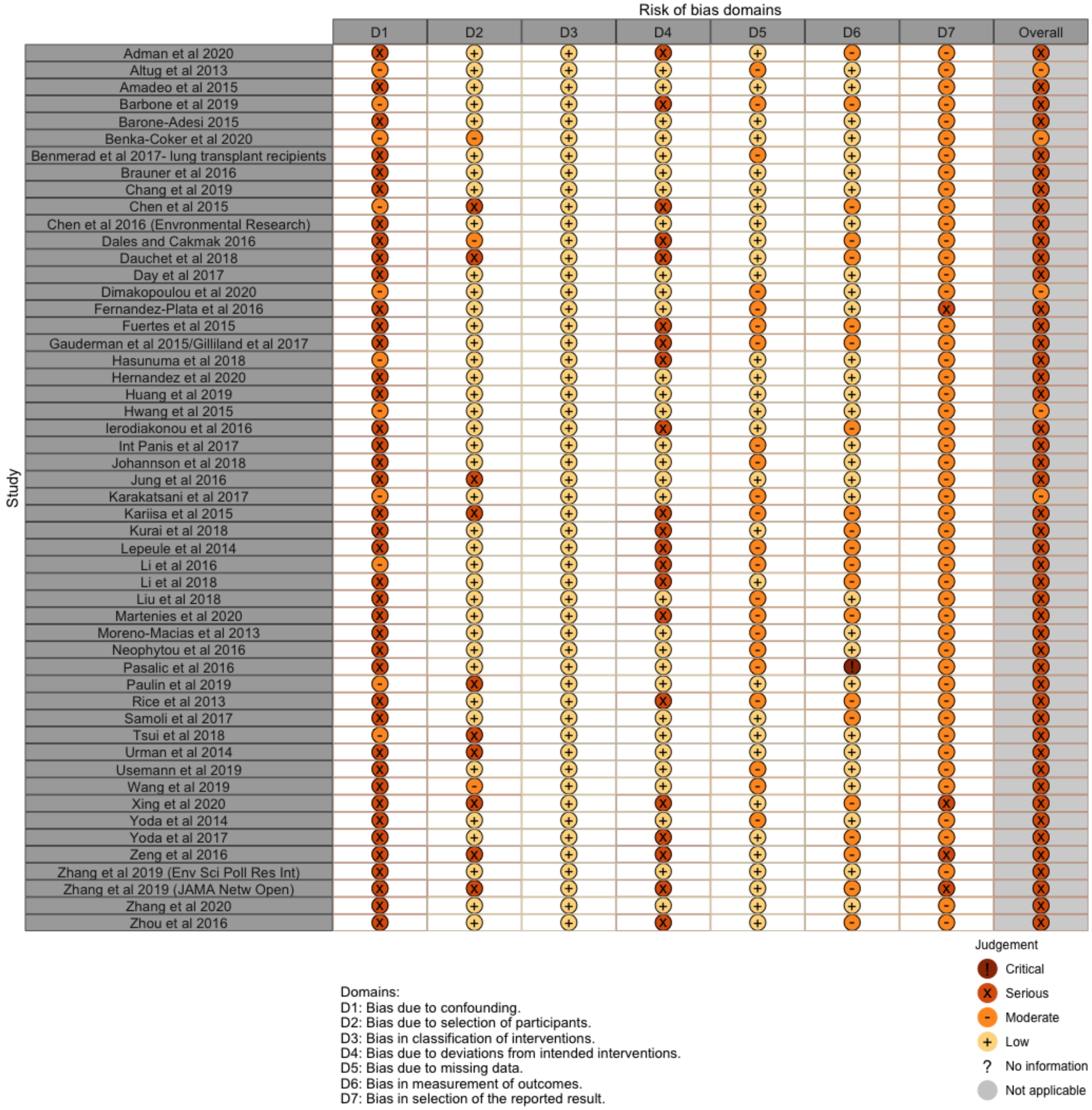

e-Appendix 5. PRISMA 2009 Checklist

Section/topic	#	Checklist item	Reported on page #
**TITLE**			
Title	1	Identify the report as a systematic review, meta-analysis, or both.	title
**ABSTRACT**			
Structured summary	2	Provide a structured summary including, as applicable: background; objectives; data sources; study eligibility criteria, participants, and interventions; study appraisal and synthesis methods; results; limitations; conclusions and implications of key findings; systematic review registration number.	1
**INTRODUCTION**			
Rationale	3	Describe the rationale for the review in the context of what is already known.	3
Objectives	4	Provide an explicit statement of questions being addressed with reference to participants, interventions, comparisons, outcomes, and study design (PICOS).	4
**METHODS**			
Protocol and registration	5	Indicate if a review protocol exists, if and where it can be accessed (e.g., Web address), and, if available, provide registration information including registration number.	NA
Eligibility criteria	6	Specify study characteristics (e.g., PICOS, length of follow-up) and report characteristics (e.g., years considered, language, publication status) used as criteria for eligibility, giving rationale.	4
Information sources	7	Describe all information sources (e.g., databases with dates of coverage, contact with study authors to identify additional studies) in the search and date last searched.	4
Search	8	Present full electronic search strategy for at least one database, including any limits used, such that it could be repeated.	4
Study selection	9	State the process for selecting studies (i.e., screening, eligibility, included in systematic review, and, if applicable, included in the meta-analysis).	5
Data collection process	10	Describe method of data extraction from reports (e.g., piloted forms, independently, in duplicate) and any processes for obtaining and confirming data from investigators.	4
Data items	11	List and define all variables for which data were sought (e.g., PICOS, funding sources) and any assumptions and simplifications made.	4-5
Risk of bias in individual studies	12	Describe methods used for assessing risk of bias of individual studies (including specification of whether this was done at the study or outcome level), and how this information is to be used in any data synthesis.	4-5
Summary measures	13	State the principal summary measures (e.g., risk ratio, difference in means).	4-5
Synthesis of results	14	Describe the methods of handling data and combining results of studies, if done, including measures of consistency (e.g., I^2) for each meta-analysis.	NA

Online supplements are not copyedited prior to posting and the author(s) take full responsibility for the accuracy of all data.
